# DIO3 protects against thyrotoxicosis-derived cranio-encephalic and cardiac congenital abnormalities

**DOI:** 10.1172/jci.insight.161214

**Published:** 2022-11-08

**Authors:** M. Elena Martinez, Ilka Pinz, Marilena Preda, Christine R. Norton, Thomas Gridley, Arturo Hernandez

**Affiliations:** 1 Center for Molecular Medicine, MaineHealth Institute for Research, MaineHealth, Scarborough, Maine, USA.; 2 Graduate School of Biomedical Sciences and Engineering, University of Maine, Orono, Maine, USA.; 3 Department of Medicine, Tufts University School of Medicine, Boston, Massachusetts, USA.

**Keywords:** Development, Endocrinology, Embryonic development, Mouse models

## Abstract

Maternal hyperthyroidism is associated with an increased incidence of congenital abnormalities at birth, but it is not clear which of these defects arise from a transient developmental excess of thyroid hormone and which depend on pregnancy stage, antithyroid drug choice, or unwanted subsequent fetal hypothyroidism. To address this issue, we studied a mouse model of comprehensive developmental thyrotoxicosis secondary to a lack of type 3 deiodinase (DIO3). *Dio3^–/–^* mice exhibited reduced neonatal viability on most genetic backgrounds and perinatal lethality on a C57BL/6 background. *Dio3^–/–^* mice exhibited severe growth retardation during the neonatal period and cartilage loss. Mice surviving after birth manifested brain and cranial dysmorphisms, severe hydrocephalus, choanal atresia, and cleft palate. These abnormalities were noticeable in C57BL/6J *Dio3^–/–^* mice at fetal stages, in addition to a thyrotoxic heart with septal defects and thin ventricular walls. Our findings stress the protecting role of DIO3 during development and support the hypothesis that human congenital abnormalities associated with hyperthyroidism during pregnancy are caused by transient thyrotoxicosis before clinical intervention. Our results also suggest thyroid hormone involvement in the etiology of idiopathic pathologies including cleft palate, choanal atresia, Chiari malformations, Kaschin-Beck disease, and Temple and other cranio-encephalic and heart syndromes.

## Graphical abstract

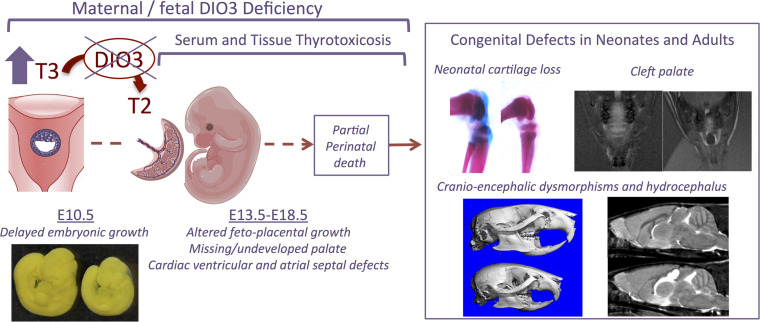

## Introduction

Human congenital defects often include abnormalities in cranial, encephalic, and cardiac tissue (1–4). This profile of tissue defects is present in multiple human syndromes that, along with variable and distinctive pathologies, usually include cleft palate, craniofacial dysmorphisms, and cardiac defects. Although genetic mutations in specific genes may explain a proportion of clinical cases for some of these syndromes, a large number of these syndromes are considered idiopathic, suggesting that other nongenetic factors may contribute to their etiology.

One of those factors could be thyroid hormones (THs). They exert a profound influence on many biological processes, including mammalian development. In particular, THs are essential for the development and maturation of the central nervous system, and a reduction in their availability may lead to severe neurological delays and marked impairments in cognitive, sensory, and motor functions (5–7). However, serum levels of THs during the early development of mammals are much lower than those in the adult, despite the fact that maternal THs can cross the placenta (8). This suggests that TH signaling needs to remain at low levels to allow for normal tissue development in the early embryo.

In humans, the importance of reduced TH action during early development is supported by the observation that maternal hyperthyroidism, whether due to Graves disease or due to genetic or environmental factors, may compromise maternal health and the viability of the fetus (9, 10). Hyperthyroidism during pregnancy is associated with increased prevalence of miscarriages (11, 12) and craniofacial and cardiac abnormalities at birth, including choanal atresia, esophageal atresia, aplasia cutis, facial dysmorphisms, aorta coarctation, and congestive heart failure (13–16). However, it is uncertain whether those defects arise from a transient excess of TH before clinical treatment normalizes maternal thyroid status or the occurrence of fetal hypo- or hyperthyroidism secondary to maternal treatment or to the transfer of maternal thyroid antibodies (13, 16–18). Currently available evidence does not support a causative effect of a specific antithyroid drug or treatment of choice (13, 16, 19, 20).

To delineate the deleterious craniofacial and cardiac abnormalities that may arise in utero from maternal hyperthyroidism, here we study a mouse model of physiological and persistent developmental thyrotoxicosis secondary to a deficiency in the type 3 deiodinase (DIO3). DIO3 inactivates the 2 main hormones secreted by the thyroid gland, thyroxine (T4) and 3,5,3′-triiodothyronine (T3). The low TH concentrations in the mammalian fetus are largely the result of the active presence of DIO3 in the pregnant uterus (21, 22), the placenta (22, 23), and most fetal tissues (24–28). Neonatal mice lacking DIO3 (*Dio3^–/–^* mice) exhibit elevated levels of T3 (29) that later in life lead to an array of severe growth (29), endocrine (29–31), and neurological abnormalities (26, 27, 30, 32), underscoring the importance of timely TH action during development. Furthermore, *Dio3^–/–^* mice exhibit impaired perinatal viability (29), although the defects underlying this outcome have not been elucidated.

Here we investigate the cranio-encephalic and cardiac abnormalities of *Dio3^–/–^* mice. We identify the occurrence of severe cranial and brain dysmorphisms, hydrocephalus, choanal atresia, cleft palate, and ventricular and atrial septal defects. This syndrome may explain the perinatal lethality in these animals and supports the notion that TH excess is the probable cause of comparable congenital abnormalities in infants born to women who developed hyperthyroidism during pregnancy. Our results raise the possibility that increased TH action during development contributes to the etiology of other comparable human syndromes that are considered largely idiopathic. Our findings also support a proactive monitoring of thyroid disease in pregnant women and prospective mothers for the prevention of congenital defects.

## Results

### Neonatal lethality and growth and cartilage defects in Dio3^–/–^ mice.

We have previously described that *Dio3^–/–^* mice exhibit reduced neonatal viability (29). Accumulated data over recent years from a large number of mice on different genetic backgrounds further illustrate this abnormality. On a 129SV/J genetic background, the genotype frequency in the offspring of heterozygous crosses showed a significant reduction in the percentage of *Dio3^–/–^* mice when surviving mice were genotyped at postnatal day 15 (P15) and at P2 (Table 1). On a mixed 129SV/J and C57BL/6J genetic background, we observed a similar postnatal reduction in the percentage of *Dio3^–/–^* mice. However, this percentage was normal when genotyping was performed in late gestation (Table 1), suggesting death occurs perinatally. We observed a similar pattern in fetal and neonatal genotype frequencies in mice on an outbred CD-1 genetic background. Interestingly, no *Dio3^–/–^* mice on a C57BL/6J genetic background survived after birth, although the anticipated proportion of *Dio3^–/–^* fetuses was present in late gestation (Table 1). These data suggest the presence of abnormalities in *Dio3^–/–^* mice that compromise life around the time of birth and that are particularly severe or prevalent on the C57BL/6 genetic background.

**Figure 1:**
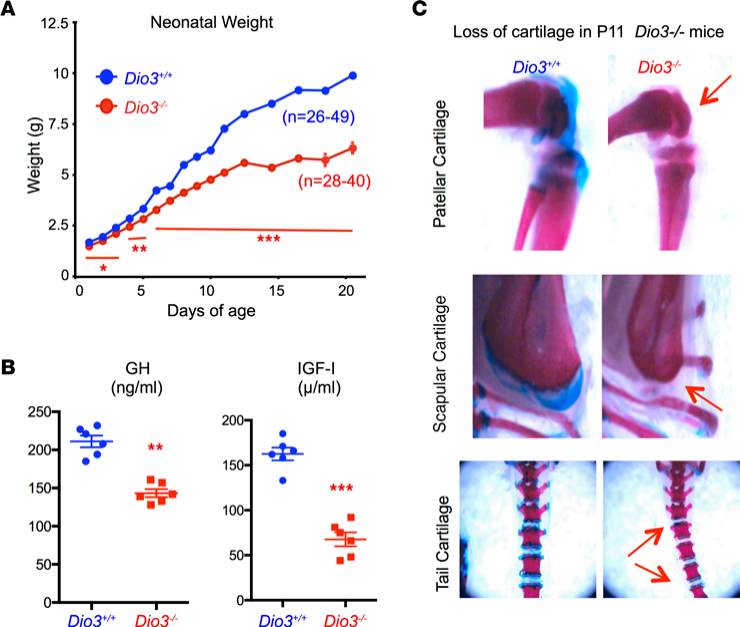
Neonatal growth and cartilage deficits in *Dio3^–/–^* mice. (**A**) Neonatal growth in *Dio3^+/+^* and Dio3^–/–^ littermates (*n* = 26–49 per data point). (**B**) P5 serum levels of growth hormone (GH) and insulin-like growth factor 1 (IGF-1). (**C**) Representative photographs taken from skeletal preparations of P11 mice showing reduced cartilage in several anatomic locations. Original magnification, 12.5×, 20×, and 10×. *, **, and *** indicate *P* < 0.05, 0.01, and 0.001, respectively, as determined by the Student’s 2-tailed *t* test. Error bars represent the standard error of the mean.

We have also described that the growth of *Dio3^–/–^* mice is delayed from weaning and into adulthood (29). Data from mice on a 129SV/J genetic background showed that this growth delay started immediately after birth, and most of it occured during the first 2 weeks of life (Figure 1A). This growth delay was associated with decreased serum levels of growth hormone (GH) and insulin-like growth hormone 1 (IGF-1) at P5 (Figure 1B). Furthermore, skeletal preparations at P11 revealed that *Dio3^–/–^* mice exhibited a marked decrease in cartilage all throughout the skeleton (Figure 1C and Supplemental Figure 1; supplemental material available online with this article; https://doi.org/10.1172/jci.insight.161214DS1).

### Craniofacial dysmorphisms in Dio3^–/–^ adult mice.

**Figure 2:**
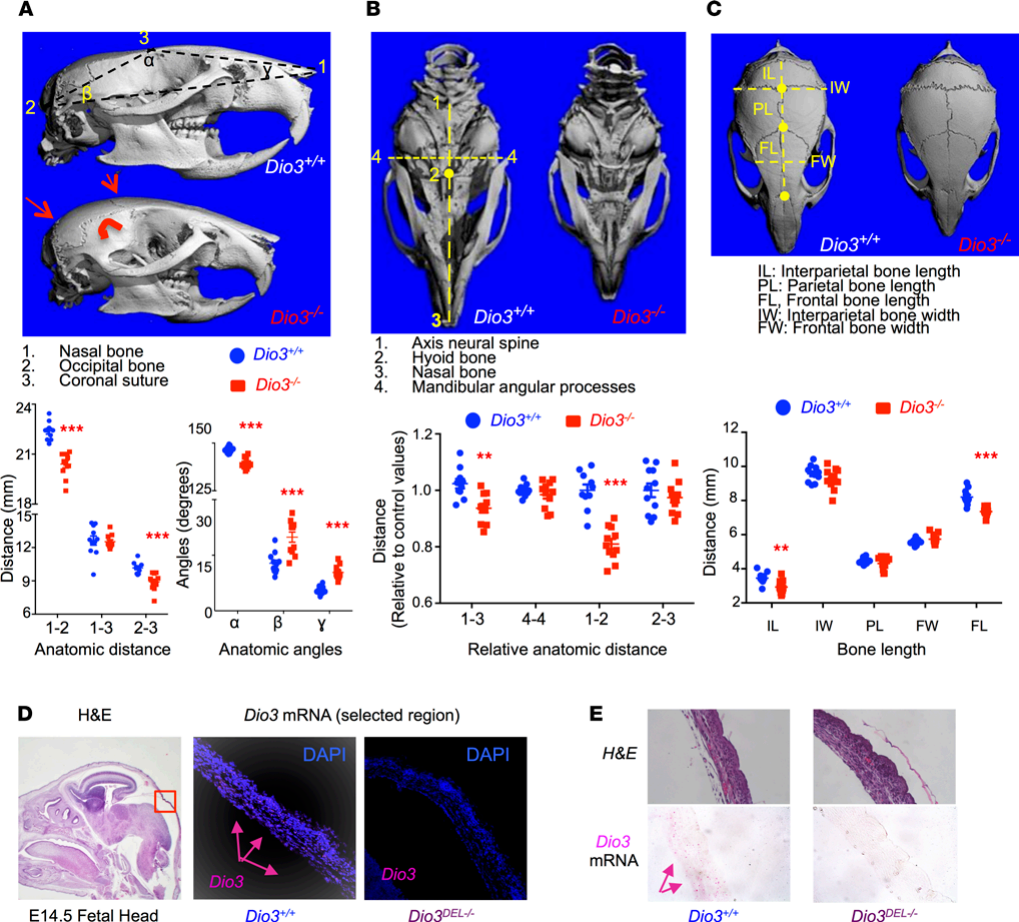
Cranial dysmorphisms in *Dio3^–/–^* mice. (**A**) Lateral-view microCT images and anatomic parameters of adult mouse crania. (**B**) Ventral-view microCT images and anatomic parameters of adult mouse crania. (**C**) Top-view microCT images and anatomic parameters of adult mouse crania. (**D** and **E**) *Dio3* mRNA expression in E14.5 fetal calvaria (pink arrows) as determined by in situ hybridization (RNAscope). MicroCT images shown represent female littermates of each genotype. Original magnification, 10× (fetal head), 200× (calvaria), 200× (**E**). **, *** indicate *P* < 0.01 and 0.001, respectively, as determined by the Student’s 2-tailed *t* test (*n* = 8–11 per genotype, including at least 4 males and 4 females).

We performed microCT scans of heads from 4-month-old *Dio3^–/–^* mice of both sexes and their *Dio3^+/+^* littermates. These mice were on a 50/50 mixed 129SV/J and C57BL/6J genetic background, being the offspring of heterozygous C57BL/6J fathers and 129SV/J mothers. The results indicated marked cranial dysmorphisms in *Dio3^–/–^* mice of both sexes (Figure 2 and Supplemental Figures 2–4). *Dio3^–/–^* mice exhibited a loss of concavity at the coronal suture; a downward rotation of temporal bones; and a markedly rounded posterior cranium involving parietal, interparietal, and occipital bones, as demonstrated by significant changes in the angles defined by the occipital and nasal bones and the coronal suture (Figure 2A). As a result, *Dio3^–/–^* crania were reduced in length, with the posterior region of the cranium accounting for most, but not all, of that reduction (Figure 2, A–C). Cranium width, as measured by the distance between mandibular angular processes, was not significantly different. We observed these abnormalities in *Dio3^–/–^* males and females, with comparable severity (Supplemental Figures 2–4).

We have previously shown that *Dio3* mRNA is strongly expressed in non-neural fetal head tissue (28). Using RNAscope in situ hybridization (Advanced Cell Diagnostics, Bio-Techne), we observed specific *Dio3* mRNA signal in the calvaria of *Dio3^+/+^* E14.5 fetuses (Figure 2, D and E) but not in littermates with a full deletion of the *Dio3* gene (*Dio3^DEL/DEL^* mice, ref. 28). Furthermore, the calvariae of *Dio3^DEL/DEL^* mice appeared thicker than that of control littermates (Figure 2E).

### Choanal atresia and palate defects in Dio3^–/–^ mice.

**Figure 3:**
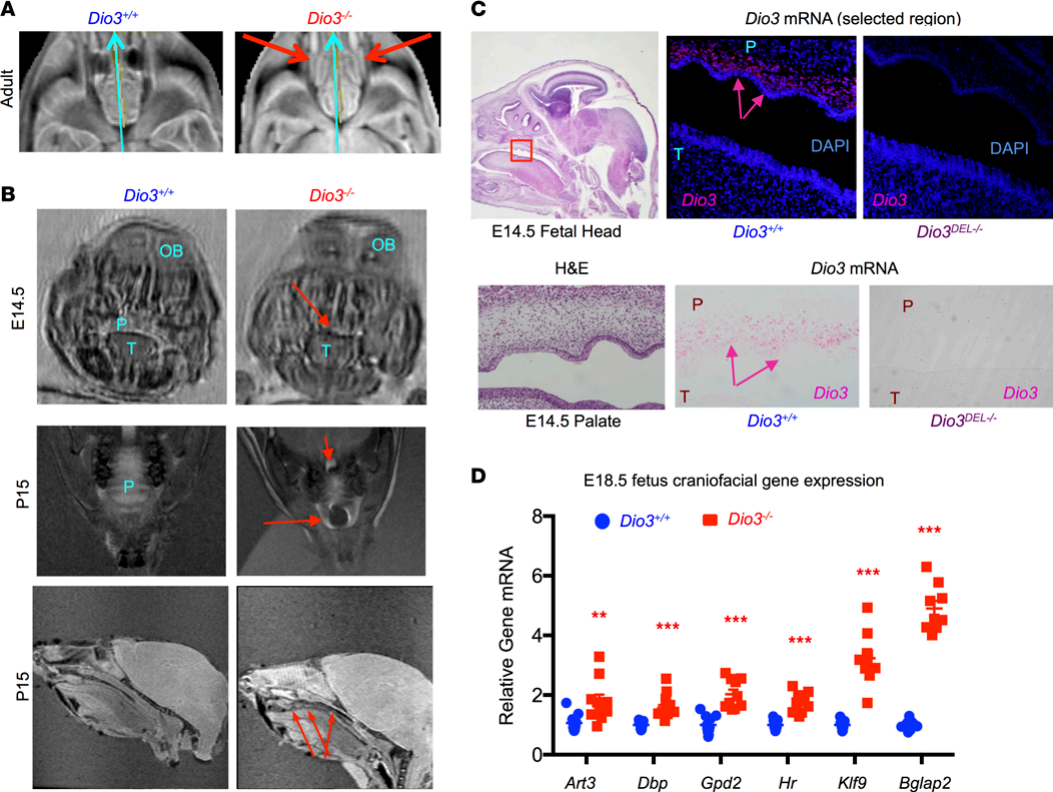
Craniofacial and palate abnormalities in *Dio3^–/–^* mice. (**A**) Representative caudo-rostral (light blue arrow) MR slice image of adult male mice (*n* = 4 *Dio3^+/+^*, 4 *Dio3^–/–^*) revealing choanal atresia (red arrows) in *Dio3^–/–^* mice. (**B**) Representative MR images of E14.5 and P15 mice showing cleft palate and palate defects (red arrows) in *Dio3^–/–^* mice (*n* = 5 E14.5 *Dio3^+/+^*, 4 E14.5 *Dio3^–/–^*, 3 P15 *Dio3^+/+^*, 3 P15 *Dio3^–/–^*). (**C**) In situ hybridization (RNAscope) demonstrating strong *Dio3* mRNA expression in the palates of E14.5 fetuses (pink arrows) (H&E image is the same as in Figure 2 and provided for anatomical reference). Original magnification, 10× (fetal head) and 200× (palate). (**D**) Expression of T3-regulated genes in E18.5 craniofacial tissue as determined by real-time quantitative PCR (qPCR). **, *** indicate *P* < 0.01 and 0.001, respectively, as determined by the Student’s 2-tailed *t* test (*n* = 11 *Dio3^+/+^*, 9 *Dio3^–/–^*). OB, olfactory bulb; P, palate; T, tongue.

Magnetic resonance (MR) imaging of *Dio3^–/–^* heads revealed additional craniofacial abnormalities. Adult *Dio3^–/–^* mice manifested choanal atresia (Figure 3A), a blockage of the nasal passage that may impair suckling during neonatal life. We also observed palate defects in *Dio3^–/–^* fetuses and neonates that varied in severity (Supplemental Figure 5A and Supplemental Figure 6). For instance, Figure 3B illustrates the lack of palate in an E14.5 fetus and a cleft palate in a P15 neonate. In situ hybridization indicated strong, specific *Dio3* mRNA expression in the developing palates of E14.5 fetuses (Figure 3C), suggesting the importance of modulating TH action in this tissue during development. Gene expression results from E18.5 craniofacial tissue of *Dio3^–/–^* mice indicated significantly elevated expression of T3-regulated genes, including *Art3*, *Dbp*, *Gpd2*, *Hr*, and *Klf9* and also osteocalcin (*Bglap2*) (Figure 3D), suggesting accelerated maturation and bone formation.

### Brain abnormalities and dysmorphisms in Dio3^–/–^ mice.

**Figure 4:**
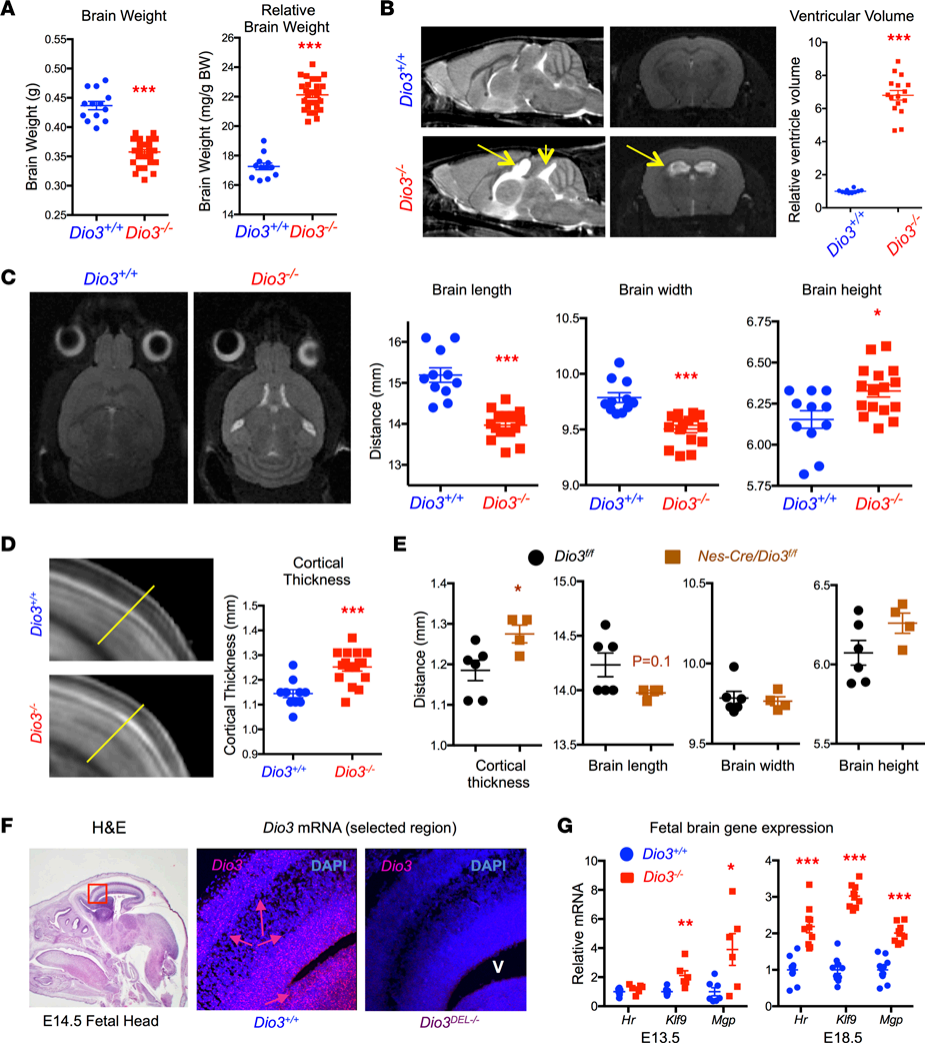
Brain dysmorphisms and hydrocephalus in *Dio3^–/–^* mice. (**A**) Brain weight and relative brain weight of adult mice (both sexes are included). (**B**) Representative rostral-caudal and coronal MR imaging of adult male brains exhibiting severe hydrocephalus (yellow arrows) in *Dio3^–/–^* mice and quantification of total ventricular volume (*n* = 10 *Dio3^+/+^*, 16 *Dio3^–/–^*). (**C**) Representative top-view MR images of adult brain and brain dimensions in *Dio3^+/+^* and *Dio3^–/–^* littermates (*n* = 10, 16). (**D**) Representative MR image and measured line and quantification of brain cortical thickness in adult mice (*n* = 10, 16). (**E**) Measurements of brain dimensions and cortical thickness in adult mice with neural tissue-specific DIO3 deficiency (*n* = 6, 4). (**F**) In situ hybridization (RNAscope) demonstrating robust *Dio3* mRNA expression in E14.5 fetal brain during corticogenesis (pink arrows), especially in the periventricular area and the newly formed outer cortex layer as determined by in situ hybridization (RNAscope) (H&E image is the same as in Figure 2 and provided for anatomical reference). Original magnification, 10× (fetal head) and 200× (brain cortex). (**G**) Expression of T3-regulated genes in E13.5 and E18.5 brains as determined by real-time qPCR. *, **, *** indicate *P* < 0.05, 0.01, and 0.001, respectively, as determined by the Student’s 2-tailed *t* test. V, ventricle.

We have previously reported that the cerebellum of *Dio3^–/–^* is maldeveloped (33). MR imaging of entire brains from 4-month-old *Dio3^–/–^* mice of both sexes on a 50/50 mixed 129SV/J and C57BL/6J genetic background revealed severe brain dysmorphisms. Consistent with growth retardation, the weight of the *Dio3^–/–^* brain was significantly reduced compared with controls, after drainage of excessive fluid. However, *Dio3^–/–^* brains were larger than *Dio3^+/+^* relative to body weight (Figure 4A). *Dio3^–/–^* brain also exhibited severe hydrocephalus, with a 7-fold increase in ventricular volume affecting the full ventricular system (Figure 4B). Brain maximum length and width were significantly reduced in *Dio3^–/–^* mice, but brain height was increased (Figure 4C).

Imaging also allowed us to examine and quantify a cortical thickness phenotype suggested by a previous observation (34). Brain cortical thickness was significantly increased in *Dio3^–/–^* mice of both sexes (Figure 4D). To determine whether this phenotype and the brain dysmorphisms of *Dio3^–/–^* mice were the result of physical constraints derived from the reduced craniofacial structure, we used a mouse model of conditional *Dio3* inactivation (*Dio3^fl/fl^*) previously described (35, 36) and crossed it with mice carrying a Cre transgene regulated by the nestin promoter. The resulting *Nes-cre/Dio3^fl/fl^* mice showed *Dio3* inactivation specific to the central nervous system as determined by DIO3 enzymatic activity in tissue homogenates (Supplemental Figure 7). The morphology of *Nes-cre/Dio3^fl/fl^* mouse brains revealed a similar profile to that of *Dio3^–/–^* mice (Figure 4E), though only the augmented cortical thickness was statistically significant because of the limited number of animals. This observation suggests that the brain dysmorphisms of *Dio3^–/–^* mice result from intrinsic DIO3 deficiency in neural tissue and are largely not secondary to cranial defects. Supporting this idea, in situ hybridization revealed that *Dio3* was expressed in the periventricular area and outer cortex of the brain at fetal age E14.5 (Figure 4F), when neurogenesis and cortical formation are particularly active. Furthermore, brain expression of T3-regulated genes was elevated in E13.5 and E18.5 *Dio3^–/–^* brains (Figure 4G).

### Congenital heart defects in Dio3^–/–^ mice and systemic thyrotoxicosis.

**Figure 5:**
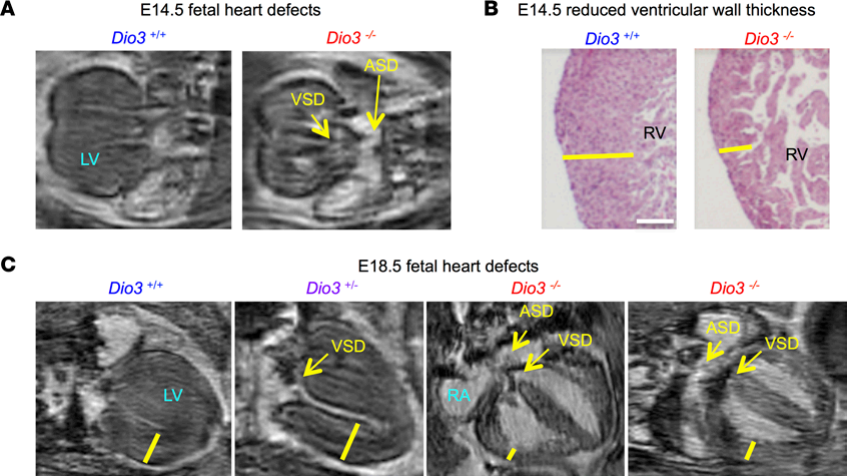
Fetal heart defects in *Dio3^–/–^* fetuses. (**A**) Representative MR images (*n* = 5 *Dio3^+/+^*, 4 *Dio3^–/–^*) illustrating congenital defects (yellow arrows) in *Dio3^–/–^* E14.5 fetal hearts. (**B**) H&E staining (*n* = 3, 4) illustrating reduced ventricular wall thickness (yellow lines) in the right ventricle of a *Dio3^–/–^* E14.5 fetal heart. (**C**) Representative MR images (*n* = 13 *Dio3^+/+^*, 6 *Dio3^+/–^*, 9 *Dio3^–/–^*) illustrating congenital defects (yellow arrows) in *Dio3^+/–^* and *Dio3^–/–^* E18.5 fetal hearts. Scale bar, 150 microns. ASD and VSD, atrial and ventricular septal defect, respectively; yellow lines indicate ventricular wall thickness; RV, right ventricle; LV, left ventricle; RA, right atria.

We performed MR imaging of E14.5 hearts from fetuses on a C57BL/6J genetic background. Four-chamber section images revealed the occurrence of both ventricular and atrial defects in *Dio3^–/–^* hearts (Figure 5A). We also observed that many *Dio3^–/–^* hearts exhibited thin ventricular walls at both E14.5 and E18.5 gestational ages (Figure 5B and Supplemental Figure 8). These abnormalities were still notable in *Dio3^–/–^* fetuses at E18.5 (Figure 5C) and were observed in some *Dio3^+/–^* littermates (Figure 5C).

**Figure 6:**
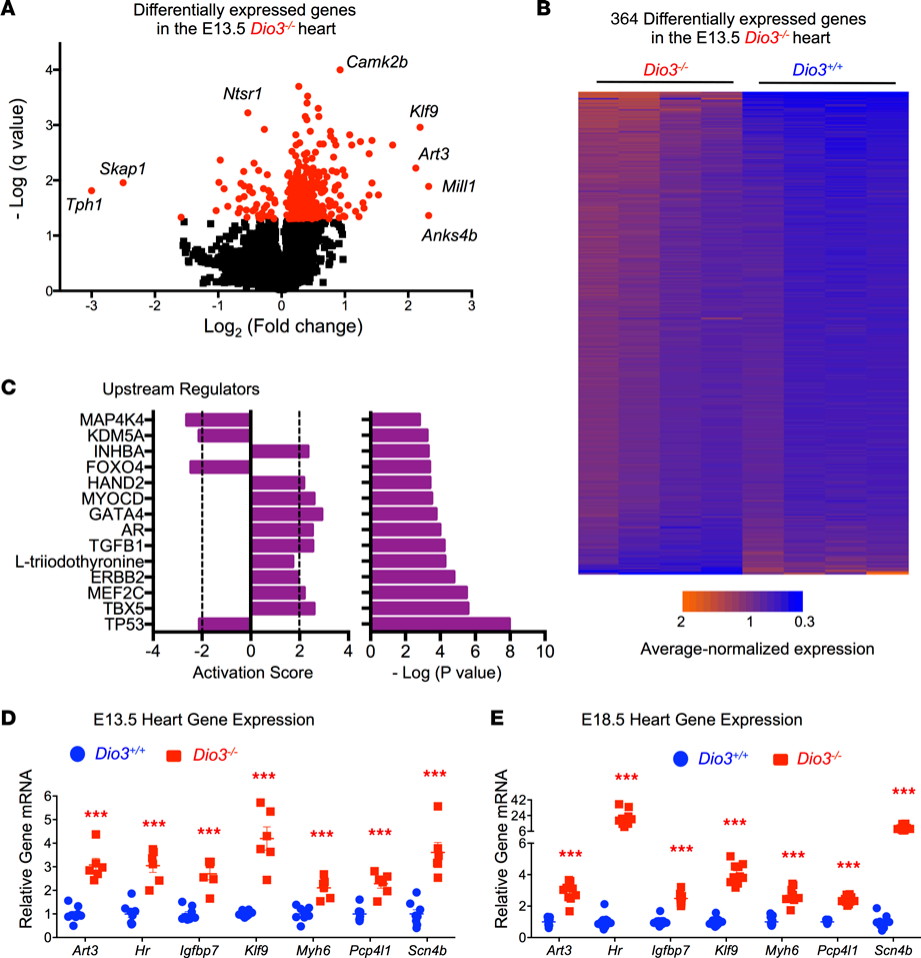
Altered gene expression in the heart of *Dio3^–/–^* fetuses. (**A**) Volcano plot of gene expression in the *Dio3^–/–^* E14.5 fetal heart. (**B**) Heatmap of 364 differentially expressed genes (*P* < 0.05) in the E14.5 fetal heart (*n* = 4, values for each gene were normalized to 1 as the mean of all samples). (**C**) Top upstream regulators (based on *P* values and activation scores) whose pathways are altered in *Dio3^–/–^* E14.5 fetal heart as determined by Ingenuity analyses. Dotted lines indicate the threshold established by the software to indicate substantial activation/inactivation (**D**) qPCR validation of differentially expressed genes in independent RNA samples isolated from E14.5 fetal hearts (*n* = 8, 6). (**E**) Expression of the same genes in E18.5 fetal hearts (*n* = 11, 8). *** indicates *P* < 0.001, as determined by the Student’s 2-tailed *t* test.

The early presence of heart defects in *Dio3^–/–^* fetuses suggested the cardiac effects of TH excess occur early during development. We used RNA sequencing to profile gene expression in the hearts of *Dio3^+/+^* and *Dio3^–/–^* E13.5 fetuses. We identified 364 genes misregulated in *Dio3^–/–^* hearts using a *q* < 0.05 cutoff (Figure 6, A and B, and Supplemental Table 1). A large percentage of abnormally expressed genes were upregulated, and the profile was largely consistent in all 4 *Dio3^–/–^* heart samples assayed (Figure 6B). Ingenuity Pathway Analysis of differentially expressed genes revealed significant upregulation of pathways dependent on TBX5, MEF2C, ERBB2, T3, TGFB1, AR, GATA4, and MYOCD and downregulation of pathways controlled by TP53, FOXO4, KDM5A, and MAP4K4 (Figure 6C). Using an extended number of samples, we confirmed the strong upregulation of *Art3*, *Hr*, *Igfbp7*, *Klf9*, *Myh6*, *Pcp4l1*, and *Scn4b* (Figure 6D). The increased cardiac expression of these 7 genes in E13.5 *Dio3^–/–^* fetal hearts of littermates was maintained or even exacerbated in *Dio3^–/–^* fetuses at E18.5 (Figure 6E), indicating marked heart thyrotoxicosis in *Dio3^–/–^* fetuses during the second half of gestation.

**Figure 7:**
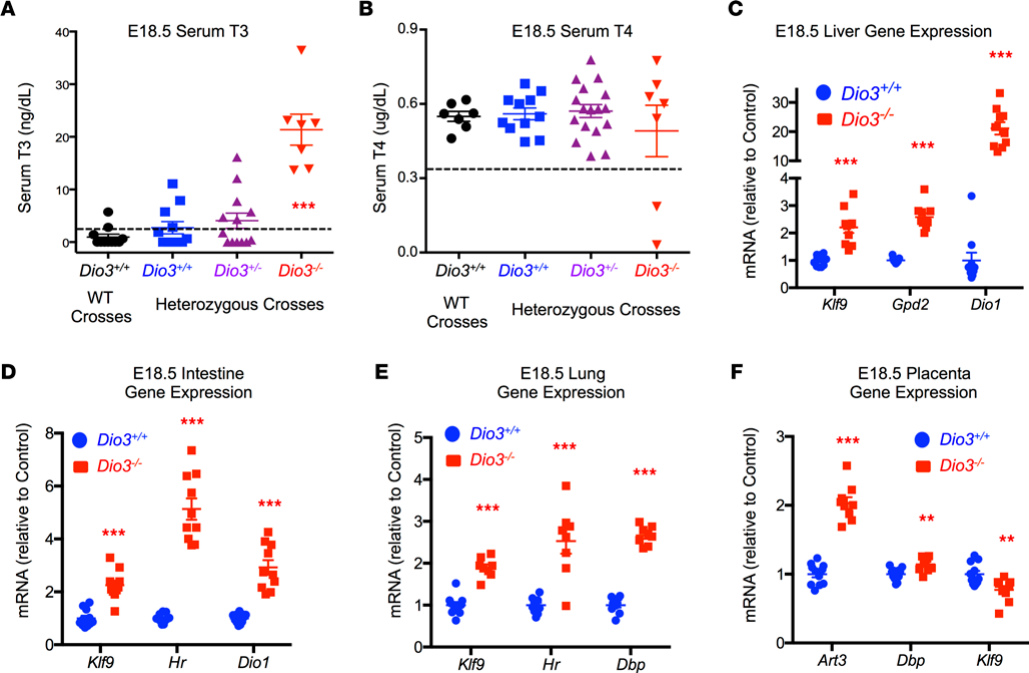
Systemic thyrotoxicosis in E18.5 *Dio3^–/–^* fetuses. (**A** and **B**) Serum T3 and T4, respectively, in E18.5 fetuses. Dotted lines indicate the sensitivity of the assays as determined by 2 standard deviations above the zeros. (**A**) *n* = 11, 11, 13, and 7. (**B**) *n* = 8, 12, 18, and 7. (**C**–**F**) Expression of selected T3-responsive genes in liver, intestine, lung, and placenta of E18.5 fetuses (*n* = 11 *Dio3^+/+^*, 8 *Dio3^–/–^*). **, *** indicate *P* < 0.01 and 0.001, respectively, as determined by 1-way ANOVA and Tukey’s post hoc test (**A**) or by the Student’s 2-tailed *t* test.

The thyrotoxicosis of *Dio3^–/–^* fetuses was systemic and extended to other tissues. Serum levels of T3 at E18.5 were significantly elevated in *Dio3^–/–^* fetuses compared with *Dio3^+/+^* and *Dio3^+/–^* littermates and with *Dio3^+/+^* generated from matings of *Dio3^+/+^* parents (Figure 7A). At this age serum levels of T4 were not significantly different among fetuses of different genotypes, independently of the parental genotypes (Figure 7B). We also analyzed gene expression in the liver, lung, intestine, and placenta of E18.5 fetuses. The expression of T3-regulated genes, including *Hr*, *Dio1*, *Gpd2*, *Dbp*, and *Klf9*, was markedly elevated in *Dio3^–/–^* fetuses compared with *Dio3^+/+^* littermates (Figure 7, C–E), indicating thyrotoxicosis in all tissues. The placenta showed increased expression of *Art3*, and *Dbp*, but decreased expression of *Klf9* (Figure 7F).

### Fetal growth and palate and cardiac defects.

**Figure 8:**
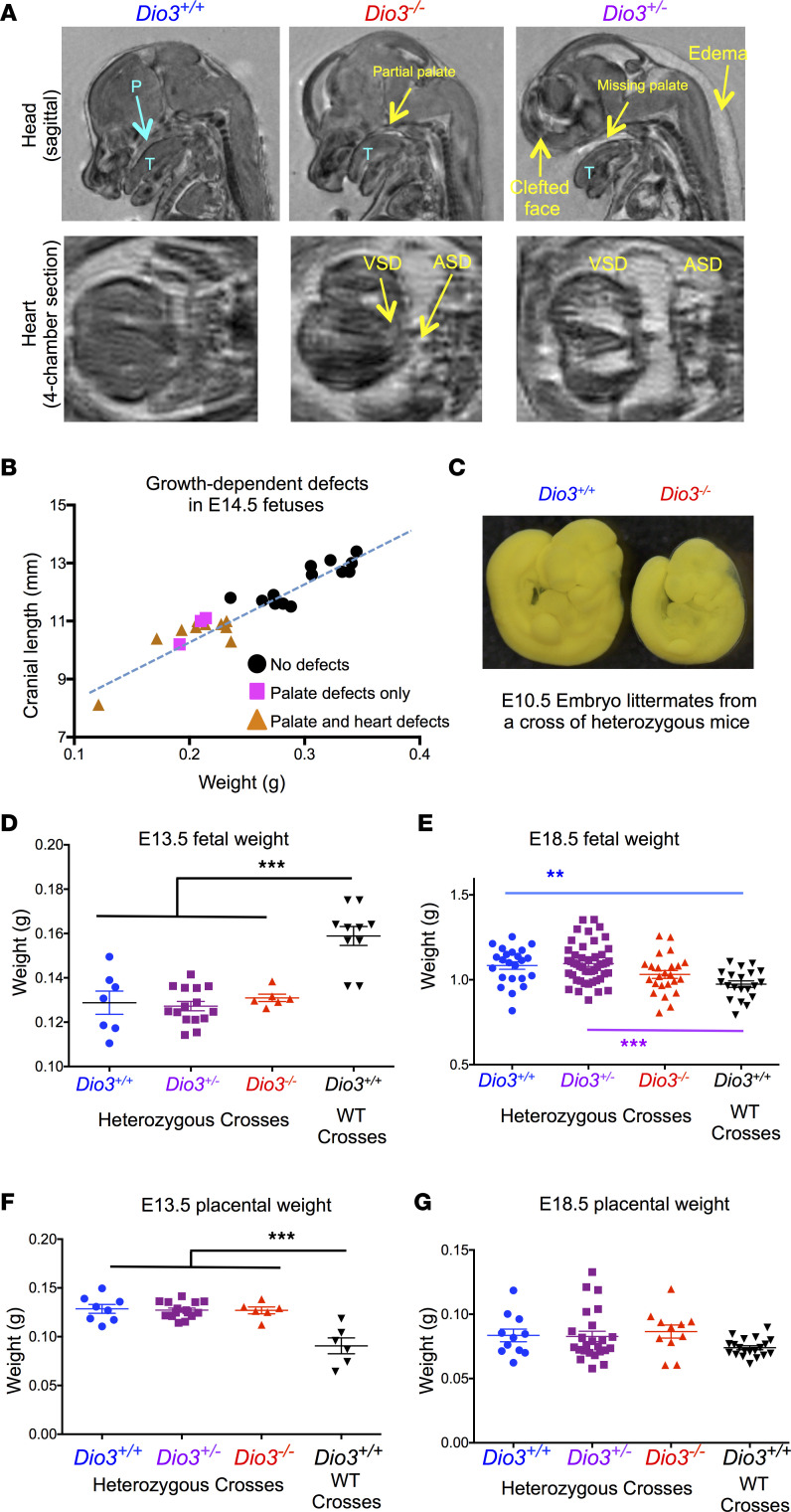
Palate and cardiac defects and altered fetal growth trajectories. (**A**) Representative MR images (*n* = 5, 10, 4) illustrating craniofacial, palate, and cardiac defects (yellow arrows) in littermate E14.5 fetuses of the 3 genotypes. (**B**) Correlation of cranial length and weight in E14.5 fetuses and association with congenital defects. Data included E14.5 fetuses of all genotypes and also fetuses generated by wild-type parents. (**C**) Photograph illustrating growth retardation in a *Dio3^–/–^* E10.5 embryos compared to a *Dio3^+/+^* littermate. (**D** and **E**) E13.5 and E18.5 weight, respectively, of fetuses of different genotypes and generated by heterozygous crosses or by WT crosses (*n* = 6–43). (**F** and **G**) E13.5 and E18.5 weight, respectively, of placentas from fetuses of different genotypes and by heterozygous crosses or by WT crosses (*n* = 6–24). **, *** indicate *P* < 0.01 and 0.001, respectively, as determined by 1-way ANOVA and Tukey’s post hoc test.

We observed that some heterozygous (*Dio3^+/–^*) littermates of *Dio3^–/–^* fetuses also exhibited palate and cardiac defects (Figure 5B and Supplemental Figures 5 and 6). Occasionally the abnormalities could also be very severe (Figure 8A). Even some *Dio3^+/+^* littermates manifested palate and cardiac abnormalities (Supplemental Figures 5, 6, and 8). However, *Dio3^+/+^* fetuses generated from *Dio3^+/+^* parents did not show abnormalities at E14.5 or at E18.5 (Supplemental Figure 9, A and B). The congenital abnormalities were more prevalent and severe in *Dio3^–/–^* fetuses than in their *Dio3^+/–^* or *Dio3^+/+^* littermates (Supplemental Figure 9, A and B). However, the occurrence of defects in fetuses of the latter genotypes suggests that the genotype of the mother and/or littermates may also be an important influence on craniofacial and cardiac development.

Weight data from E14.5 fetuses showed a strong correlation with cranial length (measured by MR imaging) for all fetuses (Figure 8B), suggesting no divergence between head and body growth. Interestingly, E14.5 fetal weights sharply separated normal fetuses from those with palate and cardiac defects, although the presence of defects was not precisely aligned with genotypes (Supplemental Figure 8A). This observation suggested retarded growth early in development. This was confirmed by embryonic size at E10.5. Figure 8C illustrates the size of an E10.5 *Dio3^–/–^* embryo compared with that of a *Dio3^+/+^* littermate. In this litter, some E10.5 *Dio3^+/–^* embryos also manifested severe growth retardation (Supplemental Figure 10). Furthermore, the E10.5 *Dio3^+/+^* embryo in this litter generated from *Dio3^+/–^* parents was smaller than any of the 6 *Dio3^+/+^* embryos of the same age generated from *Dio3^+/+^* parents (Supplemental Figure 10).

E14.5 fetal weights revealed no difference in growth between *Dio3^+/+^*, *Dio3^+/–^*, and *Dio3^–/–^* littermates, but fetuses of these genotypes were all growth retarded compared with *Dio3^+/+^* fetuses of the same age generated from crosses of *Dio3^+/+^* mice (Figure 8D). By E18.5, fetuses from heterozygous crosses were overgrown compared with *Dio3^+/+^* fetuses from *Dio3^+/+^* parents (Figure 8E). At this fetal age *Dio3^–/–^* fetuses were slightly growth retarded compared with *Dio3^+/+^* or *Dio3^+/–^* littermates and were slightly overgrown compared with *Dio3^+/+^* fetuses from wild-type crosses (not statistically significant by ANOVA and multiple comparisons test). In contrast, placental size was increased in E13.5 fetuses from heterozygous matings compared with those from fetuses from wild-type matings (Figure 8F), but we observed no differences in placental weight between *Dio3^+/+^*, *Dio3^+/–^*, and *Dio3^–/–^* littermates. By E18.5, placental sizes were similar in fetuses from *Dio3^+/–^* parents and those with *Dio3^+/+^* parents (Figure 8G). However, placentas in fetuses from wild-type parents were still smaller when compared with those from all fetuses from heterozygous parents (*P* = 0.020 by Student’s *t* test).

## Discussion

In this study we identified a syndrome characterized by several brain, craniofacial, and cardiac congenital abnormalities as the result of thyrotoxicosis in utero. We used a transgenic mouse deficient in DIO3, the enzyme that inactivates THs (37, 38) and exhibits a marked developmental pattern of expression, with high levels in fetal tissues, placenta, and pregnant uterus (22, 24, 39, 40). The *Dio3* expression profile underscores the importance of maintaining low levels of TH action during early development, despite the transport of maternal THs across the placental barrier (8). Here we show that, as expected, *Dio3^–/–^* mouse fetuses experienced profound and systemic thyrotoxicosis in late gestation. This was demonstrated by increased serum levels of T3, and by robust increases in the expression of T3-dependent genes in multiple tissues, including the liver, intestine, lung, heart, placenta, and craniofacial tissue, in addition to fetal brain, as recently described (41).

More comprehensive data than those included in the seminal work (29) showed impaired viability in *Dio3^–/–^* mice. Age-dependent genotype frequencies indicated that the death of mice with DIO3 deficiency occured either near term or immediately after birth. This incompletely penetrant lethality was consistently observed across different genetic backgrounds and reached about 100% in the commonly used C57BL/6J background. This phenotype suggests the presence of developmental abnormalities that, if sufficiently severe, are incompatible with life.

One of these abnormalities included a severely dysmorphic cranium, featuring most prominently rounded parietal, interparietal, and occipital bones; reduced cranial length; and an abnormal angle at the coronal suture. Surviving neonatal and adult *Dio3^–/–^* mice also exhibited choanal atresia and cleft palate with varying severities. These abnormalities may hamper the ability for newborns to nurse efficiently and may help explain the neonatal death of part of these animals and their retarded growth. We have previously shown that *Dio3^–/–^* mice exhibit growth delay at weaning and into adult age. Our current data showed that this growth retardation occured for the most part during the first 10 days of life, a finding that is consistent with impaired nursing and reduced neonatal serum levels of GH and IGF. These craniofacial phenotypes were equally observed in males and females and are consistent with previous literature suggesting a role for THs in craniofacial development in rats and zebrafish (42–45).

Cranial defects were accompanied by a dysmorphic brain. We have previously shown that the cerebellum of *Dio3^–/–^* mice is small and maldeveloped, with reduced foliation and accelerated maturation (33). MR image analyses of adult *Dio3^–/–^* brains of both sexes also revealed a dysmorphic brain exhibiting reduced length and width, increased height, and severe hydrocephalus. The latter may also contribute to the lethality observed in these mice and is observable at E18.5 of age (41). Consistent with initial observations (34), quantification of brain parameters demonstrated increased cortical thickness in *Dio3^–/–^* adult mice. This phenotype, together with the dysmorphic brain parameters, but notably not the maldeveloped cerebellum, were largely recapitulated in a mouse model with specific DIO3 deficiency in the central nervous system, suggesting that brain dysmorphisms are primarily due to intrinsic lack of DIO3 function in neural tissue and, to a lesser extent, to the constraints of a dysmorphic cranium.

Concerning these cranio-encephalic abnormalities, we showed strong, specific expression of *Dio3* mRNA in the fetal calvaria and palate, as well as in the subventricular, periventricular, and cortical regions of the fetal brain, where neurogenesis and cortical formation are very active at this developmental stage. Although the specific cells expressing *Dio3* remain to be identified, the discrete high *Dio3* expression underscores the importance of TH inactivation to prevent untimely TH action and the subsequent abnormal development of these structures during fetal life.

Additional abnormalities that may contribute to the reduced viability of *Dio3^–/–^* mice involve the heart. Heart imaging at E14.5 and E18.5 revealed the occurrence of ventricular and atrial defects and reduced ventricular wall thickness in most *Dio3^–/–^* fetuses. These defects were associated with marked alterations in gene expression. Gene expression profiling of E13.5 hearts revealed induction of T3-responsive genes as well as other genes relevant to heart function and to pathways related to heart development, including TP53 (46), ERBB2 (47, 48), and GATA4 (49–51). Heart gene expression at E18.5 also revealed an equally robust pattern of thyrotoxicosis. These data demonstrated a severe elevation in heart T3 signaling that may signify a premature switch to heart rate and function that normally occurs neonatally, with increased signaling through the TH receptor α (52). In most cases, cardiac defects at this age were associated with palate abnormalities, in which the anterior or the full palate was missing. Although DIO3 activity in the fetal heart was very low (data not shown), our results suggest that the tissue was affected by the systemic impairment of TH clearance as early as E13.5.

However, although palate and cardiac abnormalities were more severe and consistently shown by *Dio3^–/–^* fetuses, we also observed defects in their wild-type and heterozygous littermates. Interestingly, we observed no palate and heart abnormalities in wild-type fetuses generated by wild-type parents, suggesting that the *Dio3* genotype of the mother and/or littermates may also partially contribute to the generation of these congenital defects. The finding that, at E14.5, palate and cardiac defects seemed to be clearly correlated with retarded growth, independently of genotype, supported this idea. The substantial differences in E14.5 and E18.5 fetal and placental weights between fetuses from heterozygous parents and fetuses from wild-type parents suggest that DIO3 deficiency in maternal uterine tissue and in embryo littermates may affect the TH status in the utero-feto-placental unit, and affect early developmental processes and embryonic growth, as illustrated by the size differences in embryos at E10.5. It should be noted that we did not observe gross defects in wild-type or heterozygous animals that survived into adulthood, suggesting that DIO3-sufficient fetuses were able to ameliorate or fully correct the abnormalities exhibited in early development. Yet some mild abnormalities may persist in these mice, as those reported in the heart of adult *Dio3*-heterozygous animals (53). It is difficult to recapitulate in genetically normal animals the steady and severe thyrotoxicosis of DIO3-deficient fetuses using exogenous administration of TH. Acute administration of pharmacological doses of TH during pregnancy in rats leads to a high prevalence of perinatal death (54), consistent with the idea that developmental thyrotoxicosis may cause abnormalities incompatible with life in genetically intact rodents. In more recent work, investigators used a mouse model of T3 administration in the drinking water during the first or second half of gestation (55). Although the thyrotoxicosis achieved in this model is milder than that in *Dio3^–/–^* mice and there is a substantial latency to achieve it, the investigators observed heart hypertrophy and altered heart rate later in life (55), demonstrating that the hearts of genetically normal fetuses are sensitive to abnormal TH. Overall, our results suggest that individual fetal DIO3 deficiency is the main driver of the severe congenital defects and viability outcomes but that maternal and/or littermate DIO3 deficiency may also contribute to early developmental delays and associated miss of cranial and cardiac developmental milestones in DIO3-sufficent fetuses, possibly by affecting implantation and placental function. This hypothesis remains to be investigated.

Our findings may have important implications for multiple congenital syndromes in humans. Choanal atresia, esophageal atresia, craniofacial dysmorphisms, and cardiac defects comprise the most common congenital abnormalities whose incidence is increased in infants born to mothers who experienced hyperthyroidism during pregnancy (13–16). Our results suggest that some of the defects in these infants are possibly caused by fetal thyrotoxicosis and support the active monitoring of thyroid status in pregnant women (56, 57), and even in women before pregnancy, for prompt identification and treatment of hyperthyroidism to minimize the time the fetus might be exposed to an excess of TH. However, it is critical to note that caution is warranted when extrapolating to humans the present findings. There are substantial differences in terms of timing, source, and severity of the thyrotoxicosis, as well as in the ontogeny of THs and thyroid axis function. In terms of the latter, the second half of mouse gestation represents the second trimester of human pregnancy. With that reference, the fetal ages studied here (E10.5 to E18.5) represent the third to fifth month of pregnancy in humans, a time during which the thyroid gland is not yet significantly active. In this context, the marked elevation in serum T3 in E18.5 *Dio3^–/–^* mice appeared dramatic compared with what has been reported in human hyperthyroid fetuses, mostly during the third trimester of pregnancy. However, the observed high serum T3 in E18.5 *Dio3^–/–^* fetuses was still about 25% of adult normal values, but they appeared excessively high because of the very low baseline levels of T3 this early in development. Another important difference between mice and humans in the context of our studies is that cleft palate is very rarely observed in infants born to mothers who experience hyperthyroidism during gestation. Consistent with this observation, there were no noticeable palate defects in DIO3-sufficient mice that survived into adulthood. This suggests that the palate defects in wild-type and heretozygous mouse fetuses, which are less severe than those in *Dio3^–/–^* fetuses, are substantially corrected later in development. Thus it is possible that the palate (or other defects) of infants born to mothers who experience hyperthyroidism early in pregnancy (first trimester) follow a comparably corrective developmental course.

Cranial dysmorphism, cerebellar defects, and hydrocephalus are typical characteristics of Arnold-Chiari malformations (58). This syndrome is considered largely idiopathic, and our results suggest that unidentified TH excess during development may contribute to this syndrome. This possibility is supported by the case of a patient with an activating mutation of the thyrotropin-releasing hormone receptor. This mutation, which may have resulted in an overactive thyroid axis during late gestation, was associated with the development of a Chiari malformation (59). The use of hypervitaminosis A in developmental animal models of Chiari malformations (60) also supports the potential involvement of altered levels of TH signaling in this defect, as retinoids and THs are common partners in the regulation of gene expression through the formation of heterodimers of their respective nuclear receptors (61). Our results also show cartilage loss in *Dio3^–/–^* mouse neonates. This finding supports the involvement of DIO3-mediated alterations of TH action in the etiology of Kaschin-Beck disease, an osteoarthropathy whose severity has been associated in humans with thyroid status (62) and *DIO3* gene methylation level (63). Furthermore, a meta-analysis of genes regulating TH availability has indicated a role for DIO3 in influencing osteoarthritis susceptibility (64).

The observation that, similar to the mouse *Dio3*, the human *DIO3* gene is imprinted and preferentially expressed from the paternally inherited allele (65) also supports a role for DIO3 deficiency and subsequent TH excess in the etiology of Temple syndrome, a condition that develops as a result of maternal uniparental disomy of chromosome 14 (66, 67). As the *DLK1-DIO3*–imprinted domain is located on chromosome 14 (68), this chromosomal abnormality implies the inheritance of 2 maternal *DIO3* gene copies, which will be largely repressed and cause markedly reduced DIO3 activity. In this regard, the facial dysmorphisms, reduced head circumference, hydrocephalus, growth retardation, and neonatal failure to thrive of patients with Temple syndrome strongly overlap with the abnormalities of *Dio3^–/–^* mice, supporting a partial role for DIO3 deficiency and the associated developmental thyrotoxicosis.

In summary, we show that developmental thyrotoxicosis causes a number of craniofacial, cranio-encephalic, and cardiac defects that are probably responsible for the perinatal lethality of *Dio3^–/–^* mice. These defects are often found in multiple human syndromes, many of them considered idiopathic. Although the molecular and physiological mechanisms underlying each of these defects remain to be elucidated, our results demonstrate a critical role for *Dio3* in protecting the embryo from thyrotoxicosis, starting early in development. Our findings suggest that altered thyroid states during development, whether caused by maternal thyroid disease, particular genetic mutations, or environmental endocrine disruption, may be considered potential contributors to the etiology of cleft palate and complex craniofacial and cardiac syndromes in humans.

## Methods

### Animals.

The *Dio3^–/–^* mice used in these studies were originally created in our laboratory on a 129/SVJ genetic background. Over the years, we have backcrossed these animals for at least 7 generations into C57BL/6J inbred and CD-1 outbred genetic backgrounds. Unless otherwise indicated, the neonatal and adult animals we used in the studies were on a defined 50/50 129SV/J and C57BL/6 mixed genetic background. These animals were the F1 generation littermates generated by mating a *Dio3^–/+^* male on a C57BL/6 genetic background with a *Dio3^–/+^* female on a 129SV/J genetic background. Animals used for fetal studies were on a C57BL/6J genetic background. *Dio3* genotyping of experimental mice was performed as previously described (29). Animals were kept on a 12-hour light/12-hour dark cycle and fed regular chow ad libitum. Mice were euthanized by CO_2_ asphyxiation, followed by exsanguination, decapitation, and/or removal of vital organs. The morning on which a vaginal plug was detected was considered embryonic day 0.5 (E0.5).

### Mouse imaging.

All MR imaging was performed with a BRUKER PharmaScan 7 T, 300 MHz system. Adult mouse heads were formalin-fixed and embedded in agarose in 50 mL conical tubes. Images were obtained with a 3D RARE, T1-weighted sequence (repetition time [TR] 1,000 ms, echo time [TE] 7.13 ms, rapid acquisition with relaxation time [RARE] factor 4, averages 4, angle 180°, field of view [FOV] 28 × 29.752 × 25 mm, matrix 384 × 384 × 64, with fat suppression and motion suppression, dummy scans 2, total scan time 5 hours, 7 minutes, 12 seconds). Images were reconstructed to a matrix of 384 × 384 × 128 after acquisition.

Ex vivo fetuses (full fetus up to E14.5 and older than E14.5 heads and bodies separated) were fixed with formalin containing 4 mM gadolinium for 2 weeks and then embedded in agarose in 50 mL conical tubes. Two E14.5 and younger embryos were embedded together. A gradient echo, 3D sequence was used to obtain contrast-enhanced images: TR 45.889 ms, TE 8.5 ms, echo position 10%, averages 6, angle 50°, FOV 27 × 27 × 12 mm, matrix 512 × 512 × 256, dummy scans 2, fat suppression on, total scan time 10 hours, 1 minute, 28 seconds. Images were reconstructed to a 512 × 512 × 512 matrix and 4- and 2-chamber-view images of the heart obtained using Jive (vs. 7.9.0) with the simple slice function (69).

To examine cranium morphology and microarchitecture, we used a high-resolution desktop micro-tomographic system (vivaCT 40, Scanco Medical AG). Scans were acquired using a 17.5 μm^3^ isotropic voxel size, 55 kVp peak x-ray tube intensity, 145 mA x-ray tube current, and 300 ms integration time and were subjected to Gaussian filtration and segmentation. Skull bone structures were segmented from soft tissues using a mineral density threshold of 293.9 mg hydroxyapatite (HA)/cm^3^, 315.7 mg HA/cm^3^, 337.5 mg HA/cm^3^, or 359.2 mg HA/cm^3^. All analyses were performed using the Scanco software.

### RNA sequencing.

Total RNA from E14.5 hearts was isolated as described below and submitted to Cofactor Genomics for total RNA sequencing. Four samples were submitted per experimental group. Each RNA sample was obtained from an individual mouse. RNA samples were processed as follows and sequenced in an Illumina platform. Briefly, rRNA probes (Ribo-Zero, Epicentre) were hybridized to total RNA for removal of rRNA from the sample. Ribo-depleted RNA was then fragmented prior to cDNA synthesis using random primers. Double-stranded cDNA was end-repaired and A-tailed to prepare for adaptor ligation. Indexed adaptors were ligated to DNA, and the adaptor-ligated DNA was amplified by PCR. Library size and quality were assessed on an Agilent Bioanalyzer, and library yield was quantified by quantitative PCR using the Kapa Biosystems Library Quantification kit prior to sequencing (single-end, 75 bp fragment size) on the Illumina HiSeq 2000. The number of aligned reads per sample varied between 40 millionand 50 million and represented about 78% of the total reads per sample. Quality control, alignment, clustering, normalization, and expression comparison, analysis, and visualization were performed by Cofactor Genomics (http://cofactorgenomics.com). Raw sequence data in Fastq format were assessed for quality (FastQC, http://www.bioinformatics.babraham.ac.uk/projects/fastqc/) and rRNA content. NovoAlign version 2.08 (Novocraft, http://novocraft.com) was used to align reads to the reference genome. Parameters were trained to maximize sensitivity while maintaining the highest specificity for this data set. The resulting alignments were combined to create clusters of reads (or patches), which represent nonredundant genomic regions in the reference genome sequence. Cluster boundaries were created by taking all samples into account during the cluster generation to define the leftmost and rightmost end coordinates of each cluster. Only uniquely mapping reads were taken into consideration. While clusters represent expressed loci regardless of genomic location, clusters overlapping known genes (as defined by the reference genome assembly annotation) were labeled as such for downstream analysis. After clusters are created in this way, they undergo linear normalization by multiplying each sample’s locus coverage by the total reads of the lowest read count sample divided by the respective sample’s total reads. Mapped reads averaged 35 million per sample. These normalized expression data are the basis for the expression comparison. The comparative expression approach steps through each cluster and compares every possible pairing of samples and generates a log_2_(A/B), where A and B are the 2 normalized average coverage of each sample, A and B. Pairwise *P* values were determined among the samples using Welch’s *t* test for unequal variance. The resulting comparative expressions were visualized in ActiveSite (Cofactor Genomics), and loci of interest were chosen. *P* values for differentially expressed genes were calculated using Welch’s test, and a *q* value was calculated that adjusted for multiple testing using the Benjamini-Hochberg method. We focused our analyses on genes showing an expression level higher than 1 read per kilobase of transcript per million of mapped reads in at least 1 of the experimental groups, a fold change in expression (up or down) > 1.5, and a *q* < 0.05. Fastq files as well as processed files and metadata for this experiment have been deposited in NCBI Gene Expression Omnibus (accession number GSE198416).

Disease and functional ontology analyses were performed on selected groups of genes using Ingenuity software (QIAGEN); Functional Disease Ontology annotations (http://django.nubic.northwestern.edu/fundo/ and http://projects.bioinformatics.northwestern.edu/fundo); a service from Northwestern University (70–72); and the Database for Annotation, Visualization and Integrated Discovery (https://david.ncifcrf.gov/), a service supported by the NIH National Institute of Allergy and Infectious Diseases (73–75).

### Real-time quantitative PCR.

Fetal tissues were harvested and subsequently frozen on dry ice, and total RNA was extracted using the RNeasy kit from QIAGEN. Total RNA (1 μg) was reverse-transcribed with M-MLV reverse transcriptase in the presence of random decamers (both from Thermo Fisher Scientific) at 65°C for 5 minutes, then 37°C for 50 minutes. The 20 μL reverse transcription reactions were diluted by adding 230 μL of RNase-free water. An aliquot of each sample was mixed together for an internal standard and diluted 4-fold. Real-time PCR reactions were set up in duplicate with gene-specific primers and SYBR Select Master Mix (Thermo Fisher Scientific) and run on the CFX Connect from Bio-Rad, where they underwent an initial 10-minute denaturing step, followed by 36 cycles of a denaturing step (94°C for 30 seconds) and an annealing/extension step (60°C for 1 minute). For each individual sample, expression was corrected by the expression of control, housekeeping genes (*Gapdh* or *Rn18s*), which did not exhibit any significant difference in expression between genotypes. Expression data are shown in arbitrary units and represented as fold increase over the mean value in the control group. The sequences of the primers used for each gene are shown in Supplemental Table 2.

### RNAscope in situ hybridization and histology.

The heads of E14.5 fetuses were harvested as described above and fixed in formaldehyde for 48 hours, then embedded in paraffin and cut in 5 μm coronal or rostro-caudal sections. In situ hybridization of *Dio3* mRNA was performed in selected sections of 2 animals per genotype utilizing the RNAscope technique (Advanced Cell Diagnostics, Bio-Techne) following the manufacturer’s suggested procedures. We used the RNAscope Mm-Dio3 probe (catalog number 561641) and the ACD 2.5 HD Detection kit (RED). As a technical negative control, we use the bacterial probe DapB supplied by the manufacturer, while experimental negative controls included tissue sections from mice lacking the full *Dio3* gene (*Dio3^DEL/DEL^* mice). Some tissue sections were counterstained with hematoxylin and mounted with EcoMount (catalog EM897L, Biocare Medical), while other sections were mounted with DAPI Fluoromount-G (catalog 0100-20, Southern Biotech). Bright-field or fluorescent images of the mRNA signal were taken, respectively, with a Zeiss Axioskop 40 microscope or a Leica SP8 confocal microscope utilizing LAS X software. For anatomic reference, adjacent tissue sections were stained with H&E at our Histology Core facility following standard procedures.

### DIO3 enzymatic activity.

DIO3 enzymatic activities in tissues from *Nestin-cre/Dio3^fl/fl^* mice were determined as previously described (29). In brief, tissues were homogenized in a 10 mM Tris-HCl, 0.25 M sucrose (pH 7.4) buffer. A suitable volume of tissue homogenate was used in the enzymatic reaction to ensure that total deiodination did not exceed 40% in the assay and was proportional to the protein content. Tissue homogenates were incubated at 37°C for an hour with 2 nM ^125^I-labeled T3 (PerkinElmer) in the presence of 25 mM dithiothreitol. Deiodination was determined based on the percentage of ^125^I-3,3-diiodothyronine produced. The latter was determined by measuring the amount of radioactivity associated with the reaction products after separation by paper chromatography as described (76).

### Hormone determinations.

Fetal serum levels of T3 and T4 were determined as previously described (77–79) by highly sensitive specific radioimmunoassays using in-house–generated antibodies. We used 1 and 10 μL of serum, respectively, for T4 and T3 determinations. Neonatal serum determinations of GH and IGF-1 were performed using commercially available ELISA kits obtained, respectively, from Crystal Chem and from R&D Systems, Bio-Techne. Assays were performed according to manufacturer’s instructions.

### Skeletal preparations.

After euthanasia, the skin was removed from the mice. Mice were then eviscerated and fixed in 100% ethanol for 48 hours followed by immersion in a solution of Alcian blue, Alizarin red, acetic acid, and 70% ethanol for 72 hours. The specimens were cleared in 2% potassium hydroxide for 24 hours and brought through an ascending series of glycerol in 1% potassium hydroxide. Once cleared of tissue debris, they were transferred to 100% glycerol for long-term storage.

### Statistics.

Statistical analysis (other than RNA-sequencing data) was performed using the statistical tools of GraphPad Prism 6. A 2-tailed Student’s *t* test and 1-way ANOVA followed by Tukey’s test were used to determine statistical significance, which was defined as *P* < 0.05. Significance between genotype frequencies in the offspring of *Dio3^+/–^* matings was determined using a standard χ^2^ test. Unless otherwise stated, data are represented as the mean ± SEM.

### Study approval.

Our studies were approved by the Institutional Animal Care and Use Committee at MaineHealth Institute for Research. The animal work was performed following the guidelines of the NIH *Guide for the Care and Use of Laboratory Animals* (National Academies Press, 2011).

## Author contributions

MEM, IP, MP, CRN, TG, and AH performed experiments, generated results, interpreted data, and reviewed and edited the manuscript. MEM and AH drafted the Results section and figures, and AH conceptualized the studies and wrote the initial version of the manuscript.

## Supplementary Material

Supplemental data

## Figures and Tables

**Table 1:**
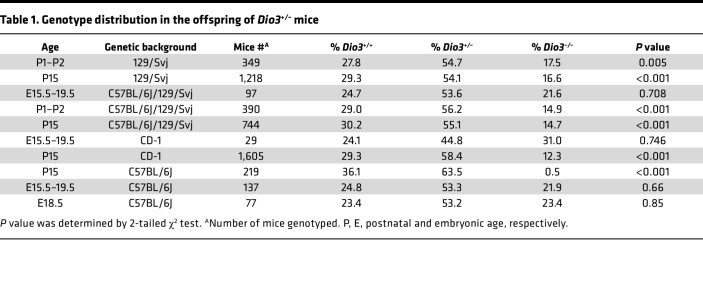
Genotype distribution in the offspring of *Dio3^+/–^* mice

## Version 1 09/27/2022

In-Press Preview

## Version 2 11/08/2022

Electronic publication
