# Supplementary material for: DIO3 protects against thyrotoxicosis-derived cranio-encephalic and cardiac congenital abnormalities
Source: JCI Insight. 2022 Nov 8;7(21):e161214. doi: 10.1172/jci.insight.161214 (PMC9675556; doi:10.1172/jci.insight.161214)
Supplement: Supplemental data [file jciinsight-7-161214-s046.pdf]

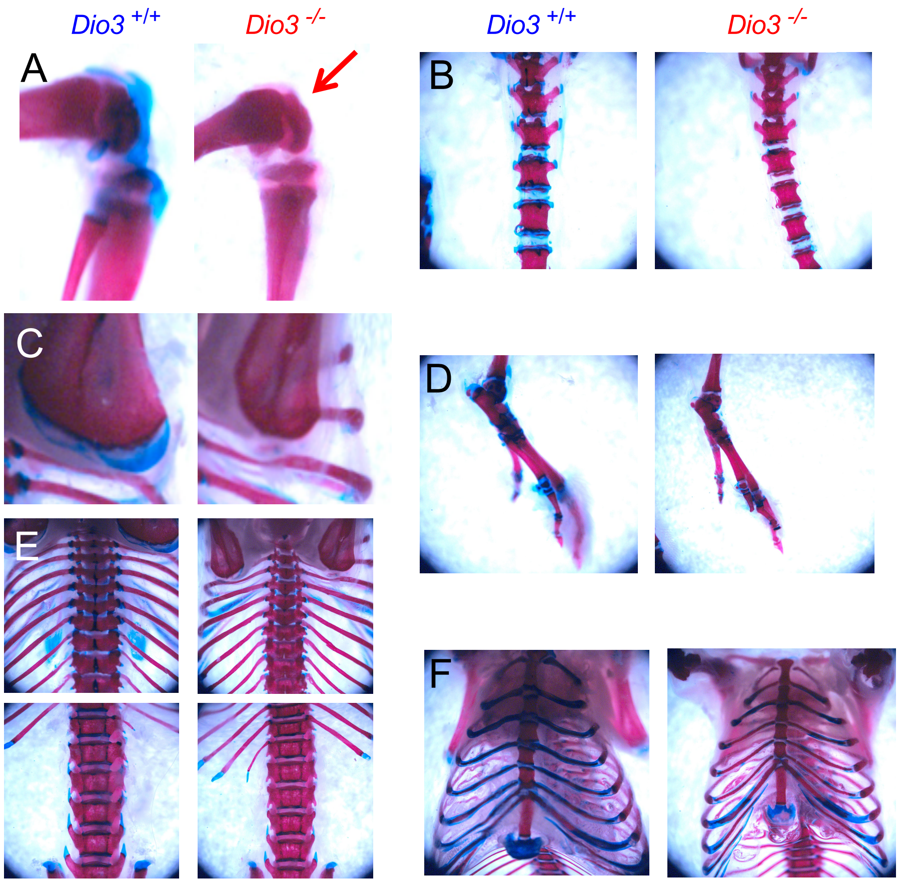

Supplemental Figure 1. .Cartilage reduction in the skeleton of P11 *Dio3 -/-* mice. Representative (n=3) photographs of *Dio3+/+* and *Dio3-/-* P11 mice illustrating loss of cartilage (Alcian blue) in DIO3 deficiency. A, Rotula; B, Tail; C, Scapula; D, Hind paws; E, Spine; F, Rib Cage.

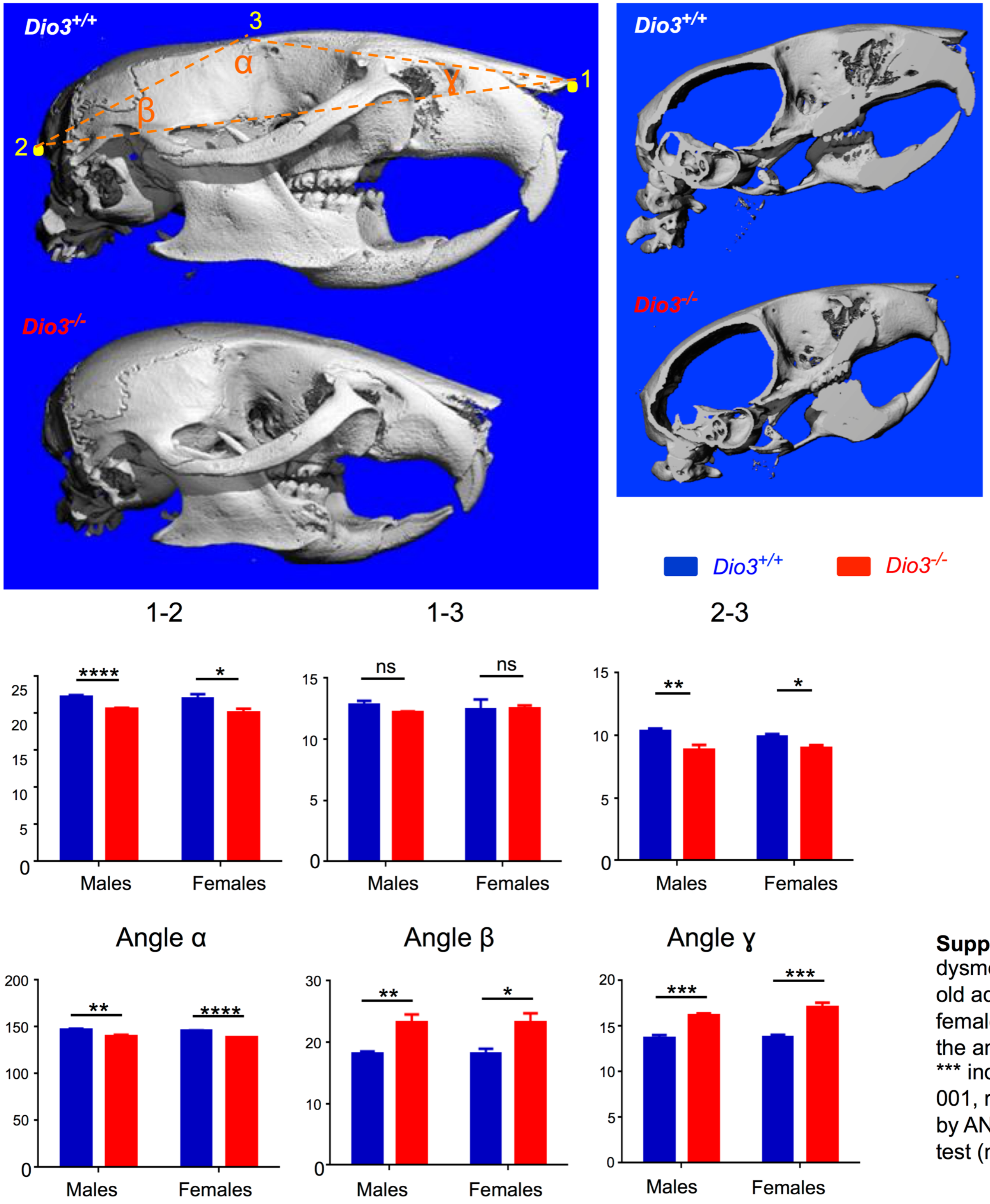

Supplemental Figure 2. .Cranial dysmorphisms in *Dio3-/-* 4-month old adult mice. Both males and females are similarly affected by the anatomic abnormalities. *, **, *** indicate P<0.05,).01 and).001, respectively, as determined by ANOVA and Tukey's post hoc test (n=4 per sex and genotype).

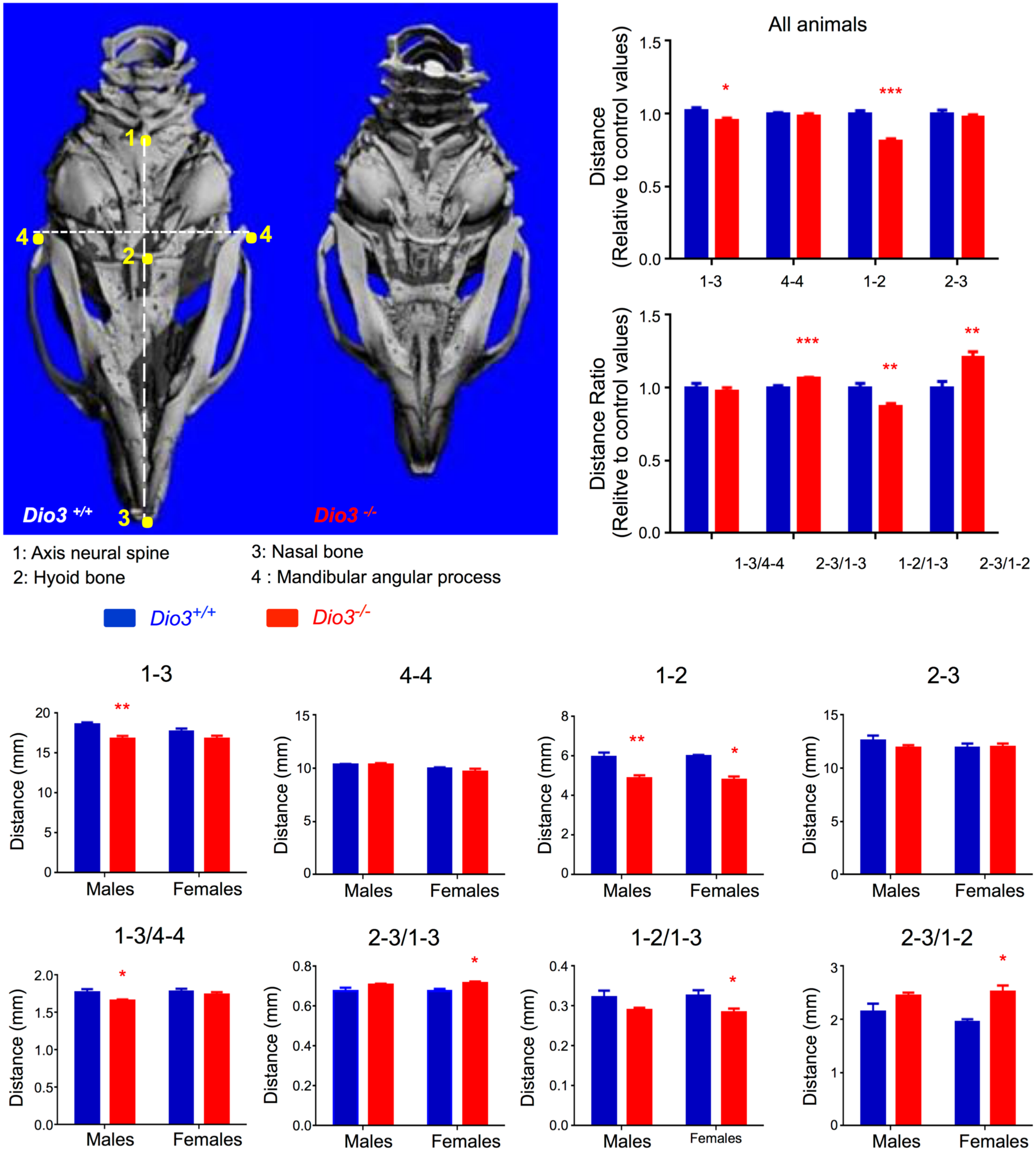

Supplemental Figure 3. .Cranial dysmorphisms in *Dio3-/-* 4-month old adult mice. Both males and females are similarly affected by the anatomic abnormalities. *, **, *** indicate P<0.05,).01 and).001, respectively, as determined by ANOVA and Tukey's post hoc test (n=4 per sex and genotype) or by Student's t-test (n=8 per genotype).

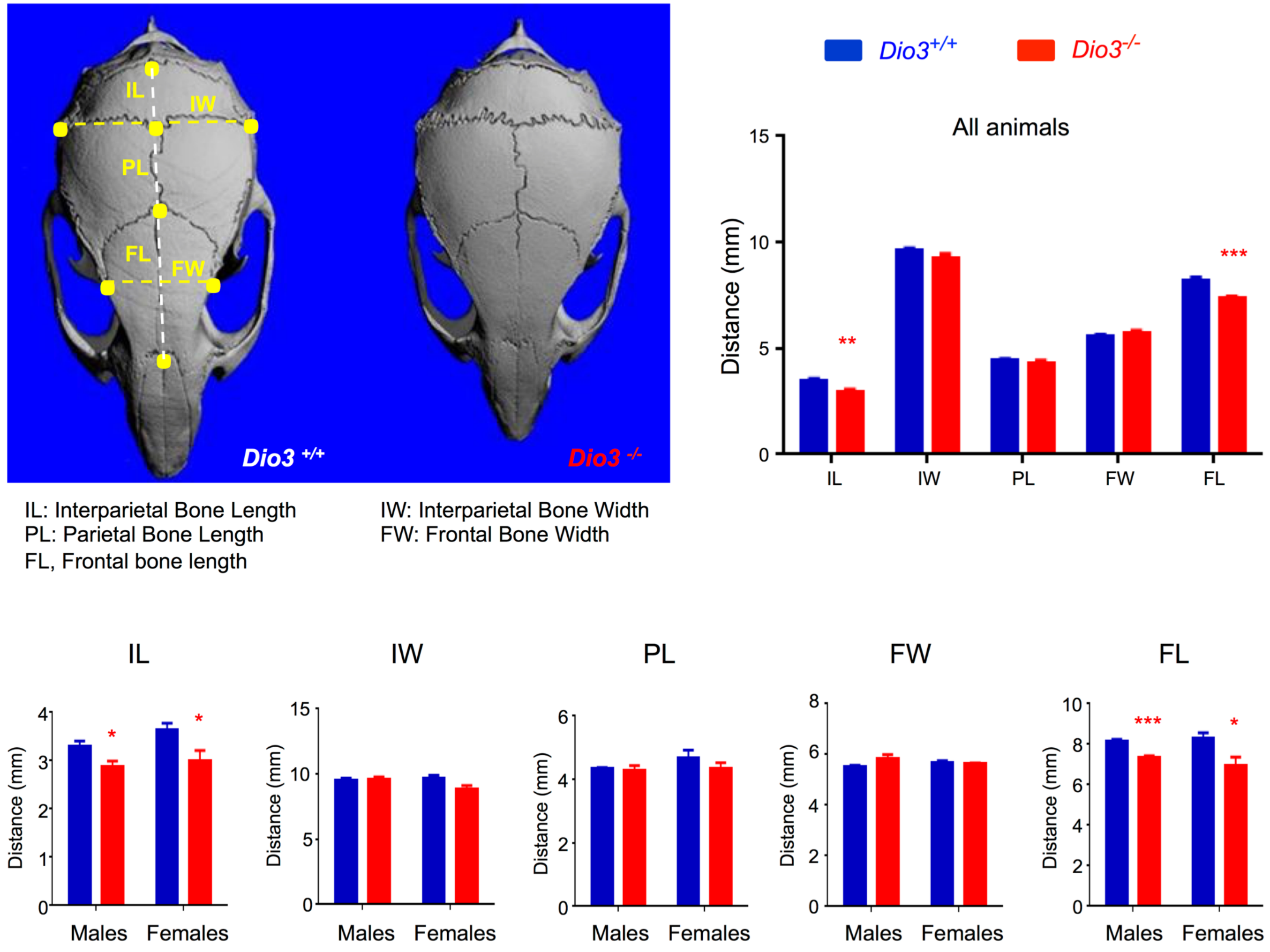

Supplemental Figure 4. .Cranial dysmorphisms in *Dio3-/-* 4-month old adult mice. Both males and females are similarly affected by the anatomic abnormalities in cranial bone lengths. *, **, *** indicate P<0.05,).01 and).001, respectively, as determined by ANOVA and Tukey's post hoc test (n=4 per sex and genotype) or by Student's t-test (n=8 per genotype).

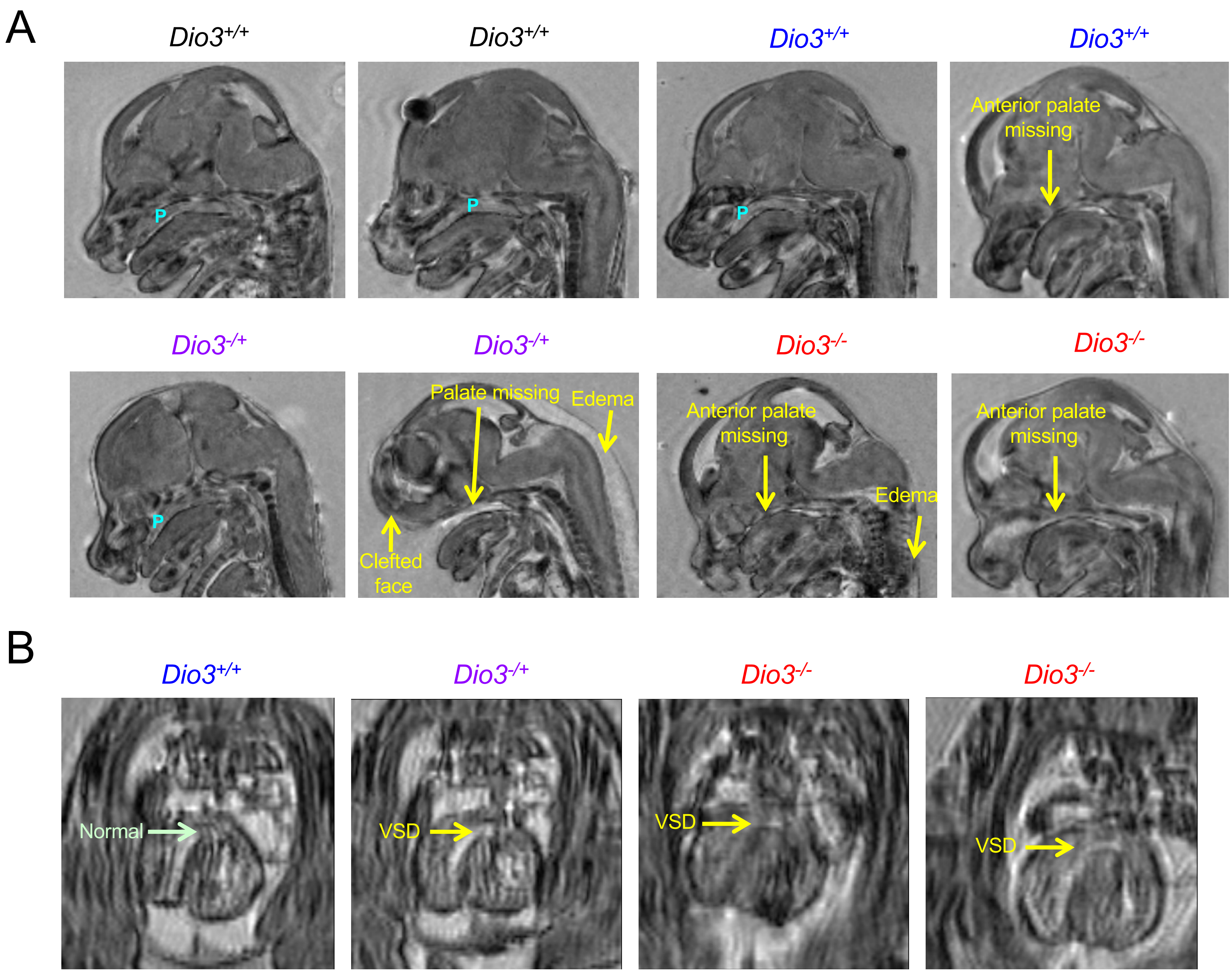

Supplemental Figure 5. .Palate (A) and cardiac defects (B) in E14.5 fetuses of different genotypes as evaluated by MR imaging. A, Palate defects and edema are indicated in yellow B, Ventricular Septal defects (VSD, yellow arrows) are observed in *Dio3-/-* mice but also in some heterozygous mice, compared to normal septum in *Dio3+/+* fetuses (green arrow). Dio3+/+ fetuses were generated by *Dio3+/+* parents. *Dio3+/+*, *Dio3+/-* and *Dio3-/-* fetuses were generated by *Dio3+/-* parents. P, palate

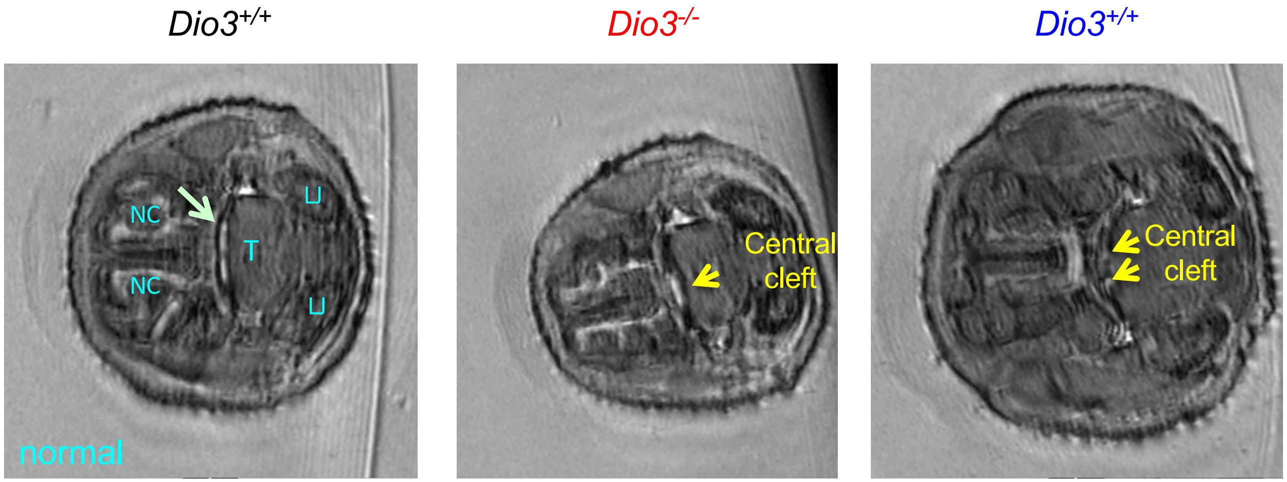

T = Tongue
NC = Nasal Chonchae
LJ = Lower Jaw
Green arrow = hard palate

Supplemental Figure 6. .Coronal MR image sections of E18.5 fetuses showing cleft palate in a *Dio3-/-* fetus and also in a *Dio3+/+* littermate.

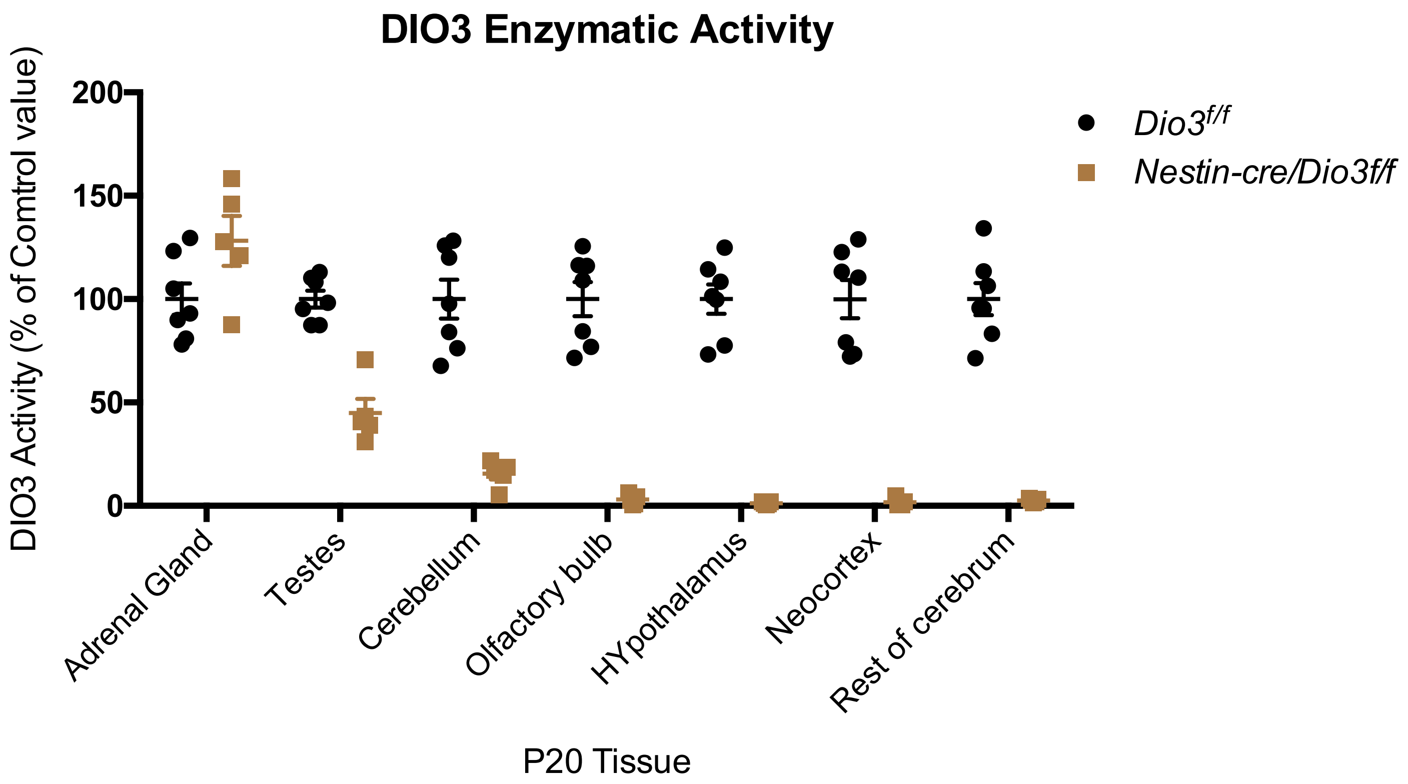

Supplemental Figure 7. Specific DIO3 inactivation in the central nervous system. DIO3 enzymatic activity in P20 tissues and brain regions of *Dio3*$^{f/f}$ and *nestin-cre/Dio3*$^{f/f}$ mice. Data represent the mean ± SEM of 7 and 5 mice for control and experimental group, respectively.

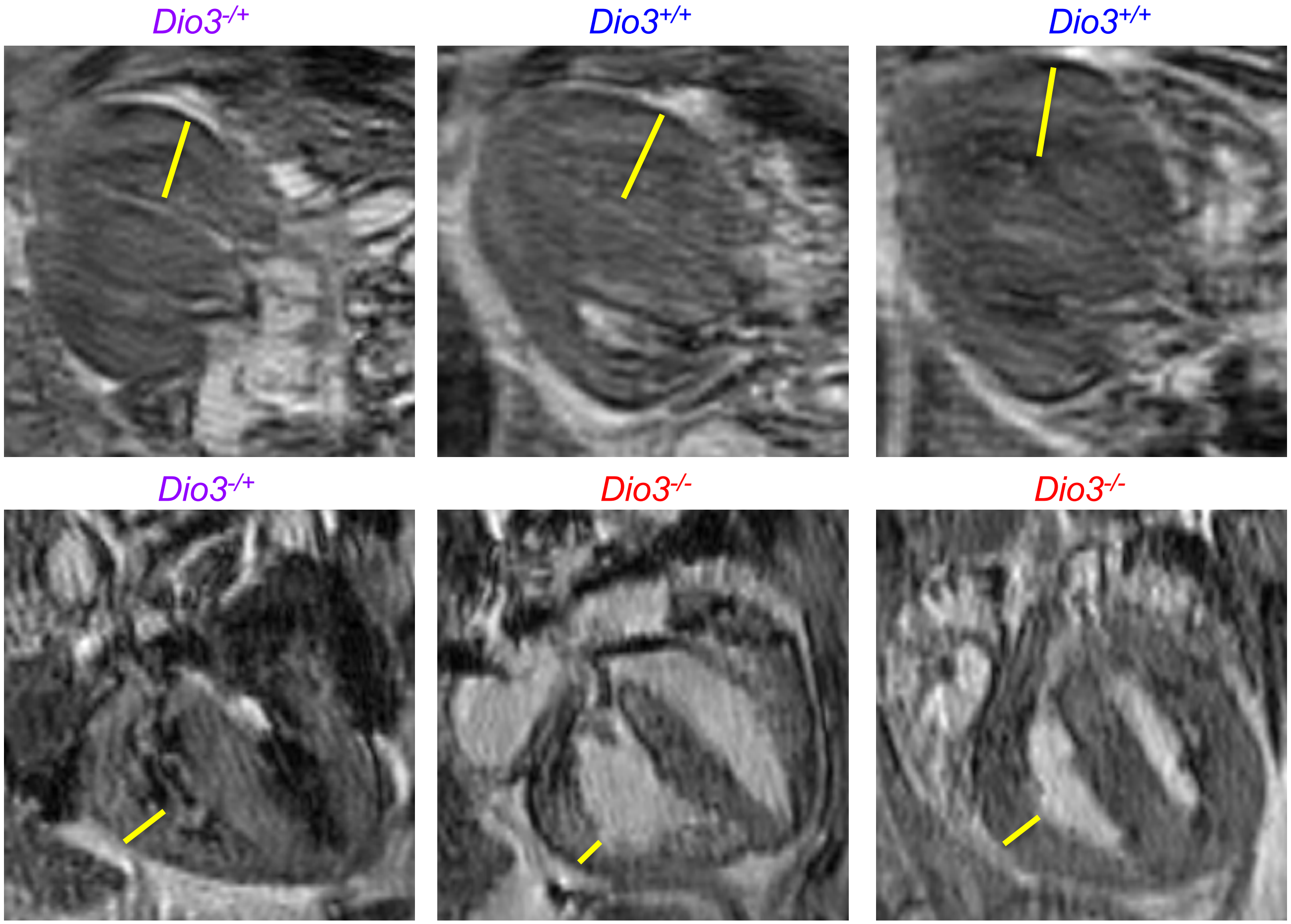

Supplemental Figure 8. Reduced ventricular wall thickness in fetal hearts. Representative MR images showing reduced ventricular wall thickness (yellow lines) in E18.5 hearts of different genotypes (bottom) versus normal hearts (top).

A

Congenital Defects in E14.5 Fetuses

Parents genotype	Fetal genotype	# of fetuses	Missing or partial palate	Ventricular septal defect	Atrial septal defect
Dio3+/-	*Dio3+/+*	5	3	2	0
Dio3+/-	*Dio3+/-*	10	5	4	3
Dio3+/-	*Dio3-/-*	4	4	4	2
Dio3+/+	*Dio3+/+*	6	0	0	0

B

Congenital Defects in E18.5 Fetuses

Parents genotype	Fetal genotype	# of fetuses	Cleft palate	Ventricular septal defect	Atrial septal defect	Heart hypertrophy or reduced ventriular wall thickness
Dio3+/-	*Dio3+/+*	13	2	2	1	2
Dio3+/-	*Dio3+/-*	6	3	2	1	1
Dio3+/-	*Dio3-/-*	9	6	3	3	6
Dio3+/+	*Dio3+/+*	7	0	0	0	0

Supplemental Figure 9. Prevalence of palate and cardiac defects in E14.5 (A) and E18.5 (B) fetuses as evaluated by magnetic resonance imaging according to fetal and parental genotype.

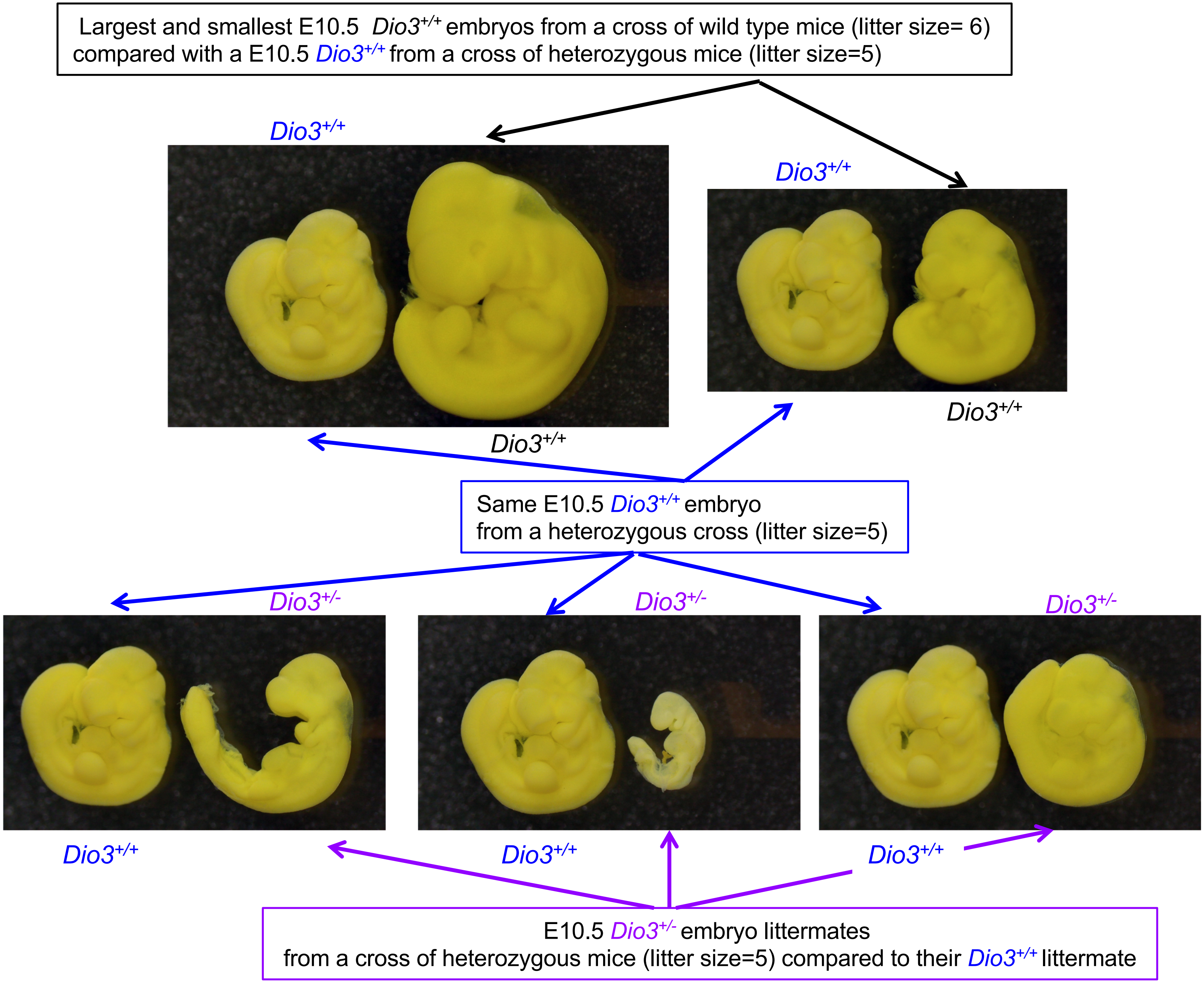

Supplemental Figure 10. Embryonic growth depends on both fetal and maternal genotypes. Relative size of E10.5 embryos as shown in pair-comparison photographs.

Supplemental Table 1. Differentially expressed genes in E13.5 fetal hearts (Dio3-/- vs Dio3+/+)

gene	q value	Log2 Fold Change
Igfbp7	0	1.764621937
Camk2b	0.0001	0.924051147
Mett11d1	0.0002	0.272079545
Osgepl1	0.0003	0.412711697
Srpr	0.0004	0.402533819
mKIAA1039	0.0005	0.579315938
Ntsr1	0.0006	-0.532495081
Gpd2	0.0007	0.3742835
Rap1gap2	0.0007	0.587921876
Letm1	0.0008	0.400331698
Klf9	0.0011	2.188761643
Sfrp1	0.0012	-0.270861486
Ehd4	0.0013	0.773212331
Ltbp4	0.0013	0.45375152
Gpld1	0.0015	0.22500837
Htra1	0.0015	1.076005564
Cldn12	0.0016	0.775233748
Atp2a2	0.0019	0.831807286
Osta	0.0019	1.427421224
Myh6	0.002	1.245111128
Ptprm	0.0021	0.286606113
Nav2	0.0022	0.659551817
Tex2	0.0022	0.604845925
Dbp	0.0023	0.856936951
Pcp4l1	0.0023	1.107589308
Slc12a4	0.0023	0.230625558
Ush1c	0.0023	1.754887502
2610029I01Ri	0.003	0.164722345
Col4a2	0.0031	0.534509557
Dot1l	0.0032	0.399930607
Guca1b	0.0033	1.38466385
Itga9	0.0034	0.359939823
Tef	0.0036	0.222392421
Bdh1	0.0038	0.822314383
Brp44l	0.004	0.482334969
Gm12238	0.0043	-0.968973104
Grip2	0.0043	0.387023123
Smarcd3	0.0047	0.263229774
Atat1	0.0049	-0.433106329
Sacs	0.0055	0.282995148
Kcnd2	0.0057	0.371968777

Ano4	0.006	0.498119241
Art3	0.006	2.119651795
Ung	0.0061	0.406370959
Lama2	0.0065	0.969339447
Tbc1d1	0.0065	0.420934242
Cdh19	0.0066	-0.362570079
Ctnna1	0.0066	0.452609902
Dnajc11	0.0066	0.252494453
Lrrc3b	0.0067	0.798165751
Abcc9	0.0069	0.666346519
Spock2	0.0069	-0.59724083
Ptprb	0.007	0.423559054
Ank	0.0071	0.581721702
Casq1	0.0071	0.86983336
Klf13	0.0072	0.395986907
Cbfa2t3	0.0073	0.236277948
Efhd2	0.0073	0.252373596
Rasa3	0.0074	0.1168024
Kcc1	0.0076	0.235535607
Alkbh7	0.0078	0.50316841
Dusp18	0.0079	-0.22450688
AK043285	0.0081	0.485426827
Dnm2	0.0081	0.096969033
.610528E23Ri	0.0082	0.475144418
Lrp8	0.0082	1
Itgb1bp2	0.0085	0.593324302
Stard10	0.0085	0.582992951
FHOS2	0.0086	0.718349916
Timm9	0.0086	0.349922881
mKIAA0348	0.0087	0.473931188
Ptpn11	0.0091	0.24880972
Qrsl1	0.0091	0.30635618
Acsl4	0.0092	0.190274433
Gpr157	0.0092	0.188381906
Arf2	0.0093	0.162245699
Atp1a3	0.0094	0.352516415
Cdkn3	0.0098	0.356200555
Mrps24	0.0102	0.206289817
Mtap6	0.0106	0.556870194
Bmpr1a	0.0108	0.472623797
Snora28	0.0109	-0.989414308
Skap1	0.011	-2.502500341
Fhod3	0.0111	0.675906238

Znrf3	0.0111	0.179978666
Ampd3	0.0112	1.426867342
Dgki	0.0113	0.652076697
Phf20l1	0.0113	0.146188221
Tcte2	0.0114	-0.641546029
Emilin2	0.0115	0.390173696
Cenpa	0.0118	0.238318662
St3gal2	0.0118	0.281691953
Alas1	0.012	0.265261881
Pdzd2	0.0122	0.894559794
Uck2	0.0122	0.314179431
Trp53inp2	0.0125	0.650138556
Synj2	0.0126	0.508772938
Nabp1	0.0127	0.427067188
Rnu1b2	0.0127	-0.59048443
Csrp2	0.0128	0.215931238
Crisp1	0.0129	#DIV/0!
Mill1	0.0129	2.321928095
Arl4d	0.013	0.482815614
Frem2	0.0131	0.852986469
Zfp874a	0.0131	-0.508464363
Nacc1	0.0133	0.167599948
Plxnb1	0.0133	0.232096685
Akap13	0.0135	0.167669733
Ank3	0.0135	0.512298367
Cep57	0.0138	0.177501592
Scel	0.0138	0.584962501
E2f6	0.0141	0.179566733
Per1	0.0141	-0.285639025
833424O15Ri	0.0142	0.644219086
Gpr37	0.0142	-0.906890596
Il1r1	0.0143	0.331843564
Wdr91	0.0144	0.126757142
Ankrd23	0.0146	0.488016945
Adarb1	0.0149	0.682557303
Irf1	0.0149	0.409112665
Pirt	0.015	0.841758216
Ehd1	0.0153	-0.205542318
Zfp874	0.0153	-0.504384985
Orai1	0.0154	0.197036847
Tph1	0.0154	-3
Ogfod2	0.0158	0.387556763
Aqp1	0.0159	0.394493922

Bara	0.0159	0.306427001
610507B11Ri	0.016	0.190583083
Tmem51	0.0161	0.655351829
BC046404	0.0163	0.215459707
Zmpste24	0.0163	0.100449929
Dennd4b	0.0166	0.265279846
Lmod3	0.0167	0.628869305
.700019L03Ri	0.0168	-0.459431619
Mtmr1	0.0169	0.238629769
Osbpl1a	0.017	0.164607284
Vegfa	0.0175	0.480955331
Ahcyl2	0.0176	-0.171368418
E2f1	0.0177	0.29180171
Loxl1	0.0179	0.244603075
Arhgef12	0.018	0.244310002
Azi2	0.0184	0.191752045
Bcng-1	0.0184	1.530514717
Pcnx	0.0185	0.176030364
Zfp385b	0.0185	1.380994036
Sgca	0.0187	0.506677744
Ccdc68	0.0188	0.931052646
Smtn	0.019	0.193935047
Actn1	0.0191	0.224118731
Sort1	0.0196	0.206730442
Suclg1	0.0196	0.291508446
AK038731	0.0197	0.464013521
C1qtnf4	0.0197	-0.589669503
Rgs7bp	0.0197	0.293820531
Usp28	0.0197	0.371485497
Bpnt1	0.0199	0.249091804
Palm2	0.02	0.371579544
Ube2r2	0.0205	-0.190988601
Pank4	0.0207	0.17692635
Vps25	0.0211	0.142590936
Ptpn4	0.0212	0.236261087
Hlf	0.0213	0.363420224
Tet1	0.0214	0.333705263
Narfl	0.0215	0.232109833
Ociad1	0.0215	0.290088373
Ache	0.0216	-0.671819373
Rtn4ip1	0.0219	0.416787281
Mrps23	0.0221	0.242464199
Rassf3	0.0225	0.289579317

Rhobtb2	0.0225	0.275815458
Cenpf	0.0227	0.232919684
Snx13	0.0227	0.181105406
Zyx	0.0227	-0.111763612
Ppme1	0.0228	0.303981183
Grsf1	0.023	0.208936984
Furin	0.0231	0.742941042
Irf5	0.0231	0.481605116
Camta1	0.0235	0.235448574
Asb18	0.0238	0.772589504
Mthfd1l	0.0252	0.245186405
Cenp-a	0.0253	0.227989266
Gsg2	0.0253	0.246837178
Lamb2	0.0253	0.42117925
AK013794	0.0254	0.544976252
Slco5a1	0.0254	1.286051059
Cdk18	0.0256	0.516453723
Dcun1d2	0.0256	0.213643486
Sin3b	0.0256	-0.103614322
Dgkz	0.0257	0.075741465
Pdp2	0.0257	0.36911478
Ank2	0.0258	0.138466559
Tgfa	0.0265	0.409800851
Tmx2	0.0265	0.27643122
Angptl3	0.0269	0.847996907
110005A03Ri	0.027	0.203315184
Ankrd32	0.0271	0.266907415
Syk	0.0271	0.380031049
Pde7b	0.0272	0.312323813
Thbs4	0.0272	-0.391989932
Tmppe	0.0272	0.155497931
Slc30a9	0.0274	0.255411165
Col4a1	0.0275	0.387113623
Wdr76	0.0275	0.281612233
Hmmr	0.0276	0.296393003
Htra2	0.0276	0.199769512
Tubb3	0.0276	0.767165832
Mmp23	0.0277	0.343720095
Gas7	0.0282	-0.235964441
Scn5a	0.0282	0.834041718
Cdkl3	0.0283	0.337500899
)830046C22Ri	0.0283	1.050626073
Arhgap11a	0.0284	0.170767943

Smad7	0.0284	0.1307746
Ppp1r11	0.0288	0.154348155
Scmh1	0.0289	0.286215362
i530418L21Ri	0.0291	0.645603852
Odf3l1	0.0295	-0.847996907
Prep	0.0295	0.281370366
Thoc6	0.0295	0.215065361
Cope	0.0296	0.136988091
Tnni3	0.0296	0.755019953
Cda	0.0297	0.496337601
Clasp2	0.0297	0.123447092
Hras1	0.0298	0.237039197
Opal1	0.0298	0.197576367
AK140072	0.0303	0.51866013
410091C18Ri	0.0305	0.108884216
Hccs	0.031	0.611823841
Sykb	0.031	0.554139577
Chpt1	0.0315	0.432959407
Mest	0.0315	-0.271220436
Slc2a4	0.0316	0.57477097
Ndufaf1	0.0317	0.161818901
Nt5e	0.0318	1.302927133
Chrm2	0.0319	0.517264997
Ranbp3	0.0324	-0.657767809
Thsd7a	0.0325	-0.415037499
Igsf1	0.0331	0.453717967
AK196015	0.0333	0.26657778
Dysf	0.0333	0.356738859
bMRP64	0.0334	0.252564501
1700040I03Ri	0.0336	0.480817574
Ndufaf4	0.0336	0.335050222
Fgf12	0.0341	0.915607813
Ctsl	0.0343	0.239209931
Phka2	0.0343	0.214720468
mKIAA0716	0.0345	0.618909833
Tnni2	0.0345	-0.554710816
Ccdc97	0.035	0.203986838
Sh3bgr	0.0351	0.657989168
2310014L17Ri	0.0353	-1.03211756
Pcca	0.0353	0.277254356
Ankrd12	0.0355	-0.453807069
Hsdl2	0.0356	0.373629402
Lrch1	0.0357	0.167319178

D19Wsu162e	0.0363	0.198276297
Upp1	0.0363	1.169533018
Tmem41b	0.0366	0.448773137
Mfsd6	0.0367	0.463246592
Scly	0.0369	0.584962501
Scml4	0.0369	0.576788569
Cd34	0.037	0.219388617
Ptcd3	0.0371	0.25264527
Arhgef10l	0.0372	0.350497247
Inpp4a	0.0377	-0.163653453
Slc7a7	0.0384	0.439322151
Kank3	0.0386	0.562419932
Srgn	0.0388	-0.684312845
Tec	0.0389	0.257365656
Dok4	0.0393	0.240675597
Klf8	0.0393	-0.308444866
AK162341	0.0394	0.244833188
)930013L23Ri	0.0395	0.398031074
Birc5	0.0396	0.202598552
Mpi	0.0397	0.2585387
Wnk1	0.0397	0.250313317
Zbtb7b	0.0397	0.391139232
Old35	0.0398	0.238699734
Prex1	0.0398	0.23878686
Acsl1	0.0402	0.491517937
Ccdc47	0.0402	0.303739599
dystrophin	0.0402	0.357261168
Heg1	0.0402	0.44127313
Ndufs4	0.0402	0.263034406
Klhdc3	0.0403	0.17749761
Qsox1	0.0403	0.356190919
Got1	0.0405	0.468374358
Cdc20	0.0406	0.151716361
Asph	0.0408	0.164953615
Mrpl21	0.0408	0.214973042
Zfp691	0.0409	0.26340485
Gtse1	0.041	0.206530892
Inha	0.041	0.787151351
Pgp	0.041	0.35630089
Rrm2	0.041	0.306311945
Stc2	0.041	0.462866024
Tpm1	0.041	0.368622372
AI414108	0.0412	0.486738965

Mecr	0.0417	0.311856884
Jmjd1c	0.0418	0.150678912
Cpsf2	0.0419	0.186645339
Mrpl2	0.0422	0.211962384
Dcaf6	0.0424	0.08302361
Trak1	0.0424	0.412957189
Zcrb1	0.0424	0.254510911
Snf2l	0.0426	-0.364233129
Gpr155	0.0427	0.373288517
Phkg2	0.043	0.158811483
Lin9	0.0431	0.253756592
Anks4b	0.0433	2.321928095
Inf2	0.0434	0.271008264
Pepd	0.0438	-0.209108265
BC057022	0.0441	0.979040381
Clptm1	0.0441	0.148043528
Mtch2	0.0441	0.676110389
Lrig1	0.0443	0.57741193
Chn1	0.0447	0.265811491
Tomm40l	0.0447	0.38332864
1300001I01Ri	0.0449	0.31886342
Agfg2	0.0449	0.252262951
Piga	0.0449	0.355331253
Trabd	0.0449	0.211251864
Ptgs1	0.045	0.317132911
Ryr2	0.0451	0.597624744
Bok	0.0452	0.375556171
Tjp1	0.0452	0.210234218
732415M23R	0.0454	1.222392421
Sox2ot	0.0454	1
Dmd	0.0461	0.392045857
Ggt5	0.0461	-0.296981738
Pcdh12	0.0461	0.154801727
Efr3a	0.0462	0.206882757
Oxct1	0.0462	0.353349532
Pear1	0.0462	0.136935645
Timm23	0.0462	0.22800239
Nceh1	0.0463	0.456638404
Slmo2	0.0463	0.263090928
Scrn1	0.0464	0.48839895
Slain2	0.0464	0.231509211
Gm6904	0.0465	-1.584962501
Pnck	0.0466	-0.545824107

Slc29a2	0.0469	0.350739006
Mthfr	0.0474	-0.357295137
Phka1	0.0474	0.125271194
Tesc	0.0475	0.910657426
Itpk1	0.0476	0.408885272
Cpeb2	0.0478	0.378365015
Dtna	0.0478	0.553865204
Slc25a4	0.0478	0.431182193
Zzef1	0.0479	0.153656612
Brd2	0.0483	-0.125187342
Alg3	0.0484	0.175127317
Opa1	0.0488	0.211585029
Fanca	0.0489	0.274243297
Mrpl54	0.0491	0.278716028
Olfml2a	0.0492	0.339126355
Arhgap44	0.0493	0.258200299
Anxa7	0.0494	-0.120072605
Clec16a	0.0495	0.167614841
Lgr6	0.0495	0.750640531
\430105I19Ri	0.0497	0.482694449
810027O10Ri	0.0498	-0.323292346
Inpp1	0.0499	0.12156198

Supplemental Table 2. Sequences of primers used in qPCR

Gene	Forward primer	Reverse primer
Art3	GACGACGAATACCTGAAGTGC	CCACTGGATCTCCGCATTGTC
Bglap2	CTGACCTCACAGATCCCAAGC	TGGTCTGATAGCTCGTCACAAG
Dbp	GGAAACAGCAAGCCCAAAGAACCGG	CAGCGGCGCAAAAAGACTCGGGC
Dio1	GGCAGAGACTGGAAGACAGG	GGCAGAGACTGGAAGACAGG
Gapdh	AGGAGCGAGACCCCACTAAC	CGGAGATGATGACCCTTTTG
Gpd2	CACTAGATGCCGTCACCAGAG	GAAGGGCTTCTTTCACCATCC
Hr	AGC ACT GTG TGG CAT GTG TT	AAC CCT GCA TCC AAG TAG CA
Igfbp7	AAGAGGCGGAAGGGTAAAGC	TGGGGTAGGTGATGCCGTT
Klf9	GGCTGTGGGAAAGTCTATGG	AAGGGCCGTTCACCTGTATG
Mgp	AGCCCAAAAGAGAGTCCAGGA	TGCCTGAAGTAGCGGTTGTAG
Myh6	TGCACTACGGAAACATGAAGTT	CGATGGAATAGTACACTTGCTGT
Pcp4l1	ATGAGCGAGCTTAACACCAAA	CTGCCAGGCTTCCCTTTTTC
Scn4b	CACCATCTTCCTCCAAGTGG	GGATGATGAGAGTCACCGTG
